# Ultrasound-Assisted Thoracic Paravertebral Block Reduces Intraoperative Opioid Requirement and Improves Analgesia after Breast Cancer Surgery: A Randomized, Controlled, Single-Center Trial

**DOI:** 10.1371/journal.pone.0142249

**Published:** 2015-11-20

**Authors:** Lijian Pei, Yidong Zhou, Gang Tan, Feng Mao, Dongsheng Yang, Jinghong Guan, Yan Lin, Xuejing Wang, Yanna Zhang, Xiaohui Zhang, Songjie Shen, Zhonghuang Xu, Qiang Sun, Yuguang Huang, The Outcomes Research Consortium

**Affiliations:** 1 Department of Anesthesiology, Peking Union Medical College Hospital, Chinese Academy of Medical Sciences, Beijing, PR China; 2 Department of Breast Surgery, Peking Union Medical College Hospital, Chinese Academy of Medical Sciences, Beijing, PR China; 3 Departments of Quantitative Health Sciences and Outcomes Research, Cleveland Clinic, Cleveland, OH, United States of America; ACTREC (Advanced Centre for Treatment, Research and Education in Cancer) / Tata Memorial Centre, INDIA

## Abstract

**Objectives:**

The contribution of ultrasound-assisted thoracic paravertebral block to postoperative analgesia remains unclear. We compared the effect of a combination of ultrasound assisted-thoracic paravertebral block and propofol general anesthesia with opioid and sevoflurane general anesthesia on volatile anesthetic, propofol and opioid consumption, and postoperative pain in patients having breast cancer surgery.

**Methods:**

Patients undergoing breast cancer surgery were randomly assigned to ultrasound-assisted paravertebral block with propofol general anesthesia (PPA group, n = 121) or fentanyl with sevoflurane general anesthesia (GA group, n = 126). Volatile anesthetic, propofol and opioid consumption, and postoperative pain intensity were compared between the groups using noninferiority and superiority tests.

**Results:**

Patients in the PPA group required less sevoflurane than those in the GA group (median [interquartile range] of 0 [0, 0] vs. 0.4 [0.3, 0.6] minimum alveolar concentration [MAC]-hours), less intraoperative fentanyl requirements (100 [50, 100] vs. 250 [200, 300]μg,), less intense postoperative pain (median visual analog scale score 2 [1, 3.5] vs. 3 [2, 4.5]), but more propofol (median 529 [424, 672] vs. 100 [100, 130] mg). Noninferiority was detected for all four outcomes; one-tailed superiority tests for each outcome were highly significant at P<0.001 in the expected directions.

**Conclusions:**

The combination of propofol anesthesia with ultrasound-assisted paravertebral block reduces intraoperative volatile anesthetic and opioid requirements, and results in less post operative pain in patients undergoing breast cancer surgery.

**Trial Registration:**

ClinicalTrial.gov NCT00418457

## Introduction

Thoracic paravertebral block (TPVB) is the technique of injection of local anesthetic adjacent to the thoracic vertebrae close to where the spinal nerves emerge from the intervertebral foramina. It appears to be a useful adjunct for breast surgery, providing effective analgesia and reducing the need for deep general anesthesia[1–5].The extent to which TPVB reduces the need for volatile anesthesia and opioids remains unclear.

Nonetheless, it is important to identify means of reducing patients’ exposure to volatile anesthetics and opioids, not least because volatile anesthetics [6,7]and exogenous opioids[8]impair numerous immune functions, including those of neutrophils, macrophages, dendritic cells, T-lymphocytes and natural killer cells (NK cells)—all of which potentially influence outcome after cancer surgery. In contrast, propofol appears to have little effect, or may even be protective, against metastasis[9,10].In an animal model, regional anesthesia and optimum postoperative analgesia had independently reduced the metastatic burden in animals inoculated with breast adenocarcinoma cells following surgery[11]. A small retrospective analysis of cancer patients suggests that paravertebral analgesia reduces recurrence risk[12].We have also undertaken a meta-analysis, which suggested that regional anesthesia was associated with improvement in prognosis of patients with operable prostate cancer[13].Nonetheless, much of this evidence is based on the findings of retrospective observational studies, in which causality could not be inferred.

Also we used a novel modified technique to perform TPVB, combining the use of ultrasound to identify the transverse process and pleura with the traditional approach. We involve in a clinical trial that has been registered at ClinicalTrial.gov (NCT00418457) since 2014, and we are the 14th centre and keeping enrollment.The results of a sub-study of an ongoing trial of regional analgesia and cancer recurrence have already been reported[14]. The protocol has been published[15]. A recent publication reported the anesthetic requirements and intensity of postoperative pain in a sub-set of the trial patients[16], but none of the patients studied here was included in the previous report.

## Methods

The study was conducted at the Peking Union Medical College Hospital, Beijing, China, between February 2014 and August 2014.The protocol was approved by the Peking Union Medical College Hospital Research Ethics Board. The exclusion criteria to select patients were: age <18 or >85 years; American Society of Anesthesiologists physical status IV or above; or any contraindication to TPVB (e.g. coagulopathy, infection, or history of allergy to local anesthetics). After obtaining written informed consent from participants, we enrolled patients scheduled for elective unilateral breast cancer resection in a prospective, randomized, double-blind, parallel-group clinical trial. The surgical procedures performed included partial mastectomy with sentinel lymph node biopsy, mastectomy, mastectomy with sentinel lymph node biopsy, modified radical mastectomy (mastectomy with axillary lymph node dissection),and mastectomy with implant insertion.

Patients were randomly assigned, in a 1:1 ratio, to receive ultrasound-assisted TPVB at the T1–T5 spinal levels followed by propofol general anesthesia(PPA group), or sham subcutaneous local anesthetic injections followed by general anesthesia with fentanyl and sevoflurane(GA group).

The randomization process was performed by the coordinating investigator using a secure password-protected web-based system controlled by the Department of Quantitative Health Sciences at the Cleveland Clinic (out of the control of any investigator). Follow-up data were collected by a research nurse blinded to each participant’s allocation.

### Preoperative procedures

After intravenous access was secured, standard routine monitoring including noninvasive blood pressure, pulse oximetry and electrocardiography was initiated. All patients were administered midazolam 1mg for anxiolysis before the block. All PPA and GA (sham blocks, only numbed the skin) were performed preoperatively at T1 to T5 with the patient in the sitting position. In both groups, the upper thoracic portion of the back, ipsilateral to the surgical side was scanned using a linear array ultrasound transducer probe (L12-3, Philips CX50) to identify the transverse processes, which were then marked on the skin. After cleaning the skin with an iodine solution, 1% lidocaine 0.2ml was infiltrated subcutaneously at each point. Then, for the PPA group, a 21–22 gauge needle with markings was used to inject 5 ml 0.75%ropivacaine into the paravertebral space at each level.

### Block technique

Participants were asked to sit in a knee-chest position, similar to the position required for neuraxial anesthesia. The patient’s feet rested on a stool to achieve a greater degree of spinal flexion while maintaining comfort. The spinous process of C7 was identified as the most prominent spinous process when the neck was flexed. The tip of the spinous process of C7 was marked on the skin.

**Fig 1:**
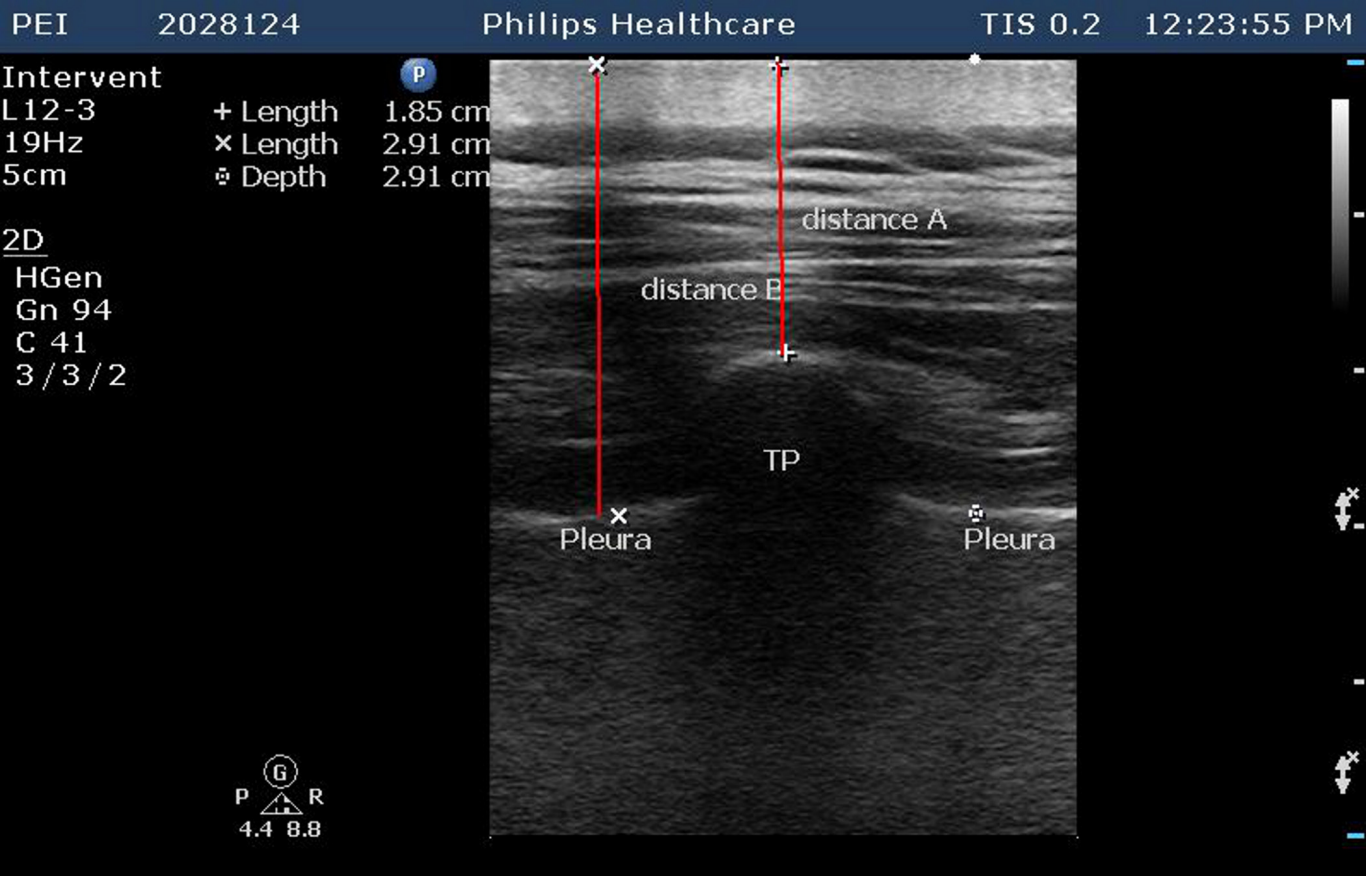
Ultrasound image of thoracic transverse process. Transverse processes (TP) appear square and lie deeper than the ribs. The distance between the skin and the TP was measured and 5 mm added (distance A). The distance between the skin and the pleura was also measured (distance B). Distance A was used as the reference for needle depth, and the needle was not advanced beyond distance B.

Scanning started 5–10 cm laterally and extended to the midline to identify the ribs and pleura. The transducer was then moved progressively more medially until the transverse processes (TPs) were identified as square structures deeper than the ribs. The midpoint of each TP was marked on the skin. We measured the distance from the skin to the TP and added 5 mm (distance A), and the distance from the skin to the pleura (distance B), without firm pressure between the skin and the transducer (Fig 1). After infiltration anesthesia, we verified the depth to the TP with a 21–22 gauge needle with markings (distance A was the target depth, and the needle was not advanced beyond distance B). After contact with the TP, the needle was withdrawn half way, and angled 10–30° caudally to walk off the TP, then the needle was advanced 1 cm further, but no more than 1.2*(distance B-distance A), and 5 ml 0.75% ropivacaine was injected at each level.

### Intraoperative procedure

The anesthesiologist providing intraoperative care was aware of group allocation. For both groups, insertion of a supraglottic airway (such as a laryngeal mask) was facilitated by rocuronium at 0.4–0.6mg/kg, and the lungs were mechanically ventilated to maintain end-tidal partial pressure of CO_2_within30–45 mmHg. Blood pressure and heart rate were maintained within 20% of their pre-operative values. If blood pressure was less than 90/60mmHg,ephedrine 6–12mg was administered. When surgery was completed, neostigmine and atropine were administered to reverse residual muscle relaxation. Ondansetron was routinely administered to prevent postoperative nausea and vomiting.

Analgesia in the PPA group was primarily based on the TPVB and maintained using a target-controlled infusion of propofol (effect site concentration 2.5–4.0 μg/ml, Marsh model). Fentanyl1–2ug/kg was given at the induction of anesthesia to facilitate insertion of the laryngeal mask. In the GA group, general anesthesia was induced with fentanyl 1–2 μg/kg and propofol 2mg/kg. Anesthesia was maintained with 2% sevoflurane and intravenous fentanyl. For both groups, additional fentanyl might be given if the blood pressure or heart rate was out of the range of 20% of pre-operative values.

Two different anesthetic regimes were selected as part of our study design. The potential benefit of perioperative use of regional anesthesia combined with propofol sedation may reduce the risk of cancer recurrence and metastasis, owing to its more favorable influences on the immune system than volatile anesthetics, opioids and surgical stress. We chose to compare a propofol–regional anesthesia approach with a traditional sevoflurane–fentanyl anesthetic regimen that is in widespread clinical use, and judged that using either propofol or sevoflurane would have little impact on intraoperative fentanyl consumption or postoperative analgesia when bispectral index(BIS) was used to titrate the depth of anesthesia. While it might be possible to independent control volatile anesthetic and opioid use, thus isolating the “pure” effect of paravertebral blocks, that approach would be highly non-physiological. We thus considered the overall consequence of a putative clinical decision to use a block.

### Postoperative procedure

At the end of the procedure, all patients were transferred to the post-anesthesia care unit (PACU) where they were monitored until they met the requirements to the ward. PACU nursing staff, who were unaware of the patient allocation, monitored patients for pain and postoperative nausea and vomiting.

### Measurements

The primary outcomes included volatile anesthetic dose was measured in MAC-hours; intraoperative fentanyl and propofol doses; the intensity of the most severe surgical site pain in the first two postoperative hours on a 10-cmvisual analog scale (VAS).

Secondary outcomes included intraoperative ephedrine use, core temperature at the end of surgery, time-weighted mean arterial pressure (MAP), time-weighted heart rate, and nausea or emesis in the PACU.

### Statistical analysis

We planned *a priori* to claim regional intervention more effective than general anesthesia alone if it could be shown to be noninferior to general anesthesia for all four primary outcomes, and superior to general anesthesia for at least one of the four. [17, 18]

Noninferiority was tested at the 0.025 level for each outcome in one-tailed tests. If noninferiority was claimed on all four outcomes, superiority would be tested on each outcome with one-tailed tests (Wilcoxon rank sum test) in the hypothesized direction at the overall 0.025 level, adjusting for multiple comparisons with the Bonferroni correction (0.025/4 = 0.00625 one-tailed significance criterion).

We a priori defined the noninferiority delta as 1 cm on the visual analog pain score, and used the difference between medians as the main outcome measure for pain score. The noninferiority delta for intraoperative fentanyl or sevoflurane consumption was a priori defined as a ratio of mean ranks of 1.1, since lower values for these parameters were desirable. While for propofol (mg), the delta was a ratio of mean ranks of 0.9 since higher values were desirable.

We used a ratio of mean ranks instead of ratio of medians or means because these variables were not normally or even log-normally distributed. Furthermore, the distributions did not have similar shapes between the two groups, making a comparison of medians inappropriate and potentially disjoint from the Wilcoxon rank-sum test results. For example: a ratio of mean ranks of 1.1 indicates that when an outcome variable is ranked from smallest to largest across all patients, the average ranking (or equivalently, average percentile) for the regional group is 10% higher than the average ranking for the general-opioid group; or that regional anesthesia increased the mean ranking by 10%. Likewise, a ratio of 0.5 would indicate that patients in the regional group had a 50% reduction in the mean rankings for that outcome.

The percentile bootstrap resampling method was used to estimate the 99.75% confidence interval for difference in medians and ratio of mean ranks for all outcome measures comparing the regional and general groups. Noninferiority was claimed if the upper confidence limit was below the noninferiority delta (except for propofol, for which the lower confidence limit needed to be above the noninferiority delta).

To have 90% power at the 0.025 significance level to detect superiority on at least one of the 4 outcomes with a 1-tailed test, a total sample size of 50 per group would be needed. This a-priori assumes variability equal to what was observed in the current dataset, and differences of 20% of the control mean for each of opioid consumption, sevoflurane and propofol, plus a difference of 2 for VAS pain, and adjusts for multiple comparisons.

The t-test or Wilcoxon rank sum test was used to assess continuous secondary outcomes, and the chi-square test for binary outcomes. Results are presented as the mean ± standard deviation, median [first, third quartiles], or median difference ratio of mean ranks (with 98.75% CIs). The SAS statistical software program (Cary, NC) was used for all analyses.

## Results

**Fig 2:**
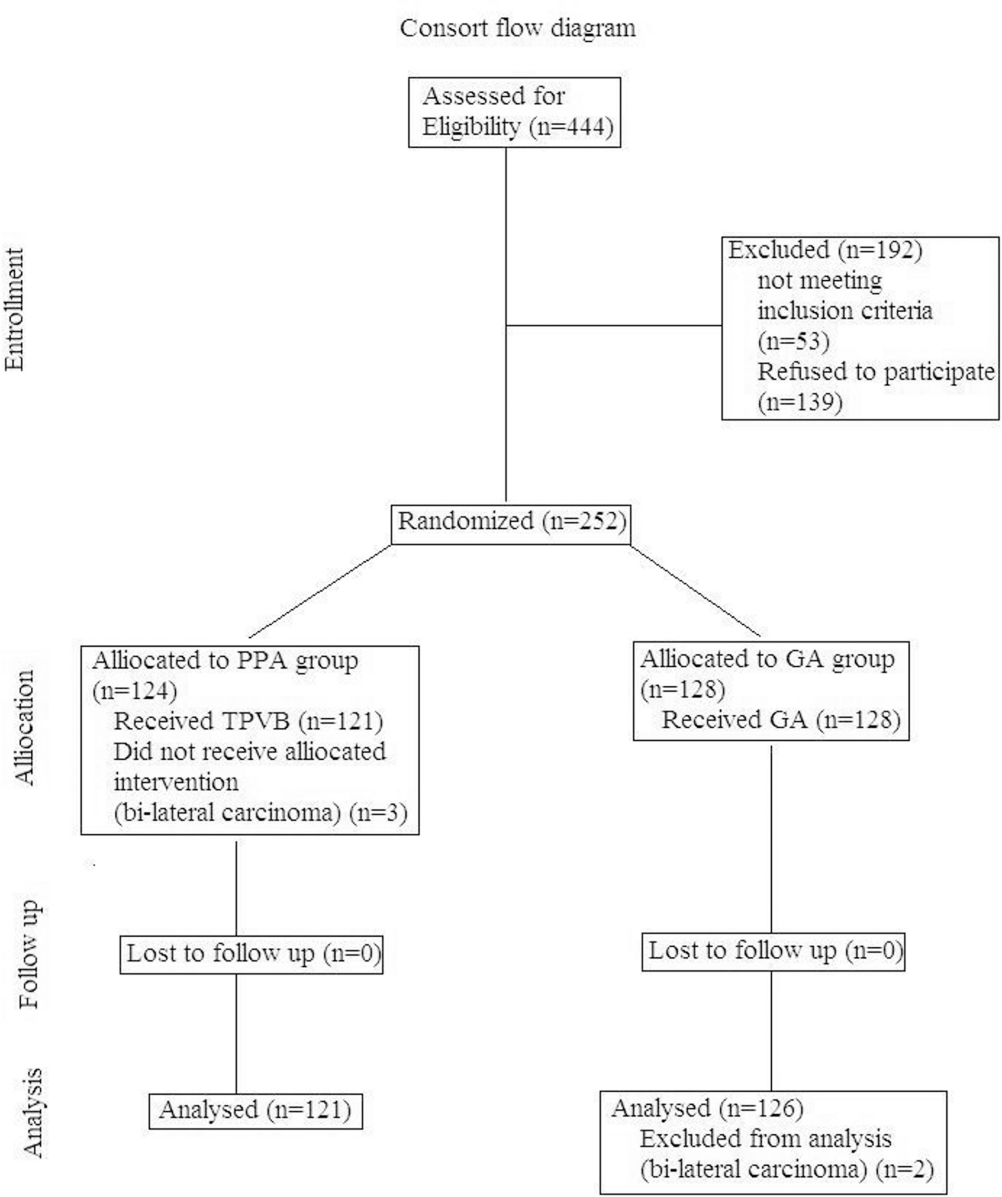
Consolidated Standards of Reporting Trials (CONSORT) flow diagram depicting subject progress through the phases of the study. PPA group: thoracic ultrasound-assisted thoracic paravertebral blocks at the T1–T5 thoracic levels with propofol-based general anesthesia; GA group: sham subcutaneous local anesthetic injections with sevoflurane-based general anesthesia and opioid-based analgesia (GA group).

A total of 247 patients randomized to treatment allocation completed the study (n = 121 in the PPA group and n = 126 in the GA group); all the data were included in the analysis (Fig 2). Morphometric, demographic characteristics, and surgical factors were well balanced between two groups (Table 1).

**Table 1:** Demographic and morphometric characteristics of participants. Data are reported as number (%) or mean ± standard deviation. Abbreviations: ASA, American Society of Anesthesiologists; BMI, body mass index; STD, standardized difference–we considered as imbalanced any variable with absolute STD ≥ 1.96 $\sqrt{2/Npergroup}$ = 0.25.

Factors	PPA group	GA group	STD
	(N = 121)	(N = 126)	
**Age (yr)**	46 ± 13	46 ± 12	0.01
**BMI (kg/m** ^**2**^ **)**	23.7 ± 3.3	23.5 ± 3.2	0.05
**ASA status**			-0.09
** I**	84 (69)	82 (65)	
** II**	37 (31)	44 (35)	
**Menopausal status**			0.12
** Pre-**	61 (50)	70 (56)	
**Peri-**	15 (12)	12 (10)	
** Post-**	45 (37)	44 (35)	
**Type of surgery**			0.12
** Simple mastectomy**	0 (0)	0 (0)	
** Modified radical mastectomy**	98 (81)	96 (76)	
** Wide local excision with node dissection**	13 (11)	17 (13)	
** Other**	10 (8)	13 (10)	
**Duration of surgery (minutes)**	67 ± 29	65 ± 26	0.09

**Fig 3:**
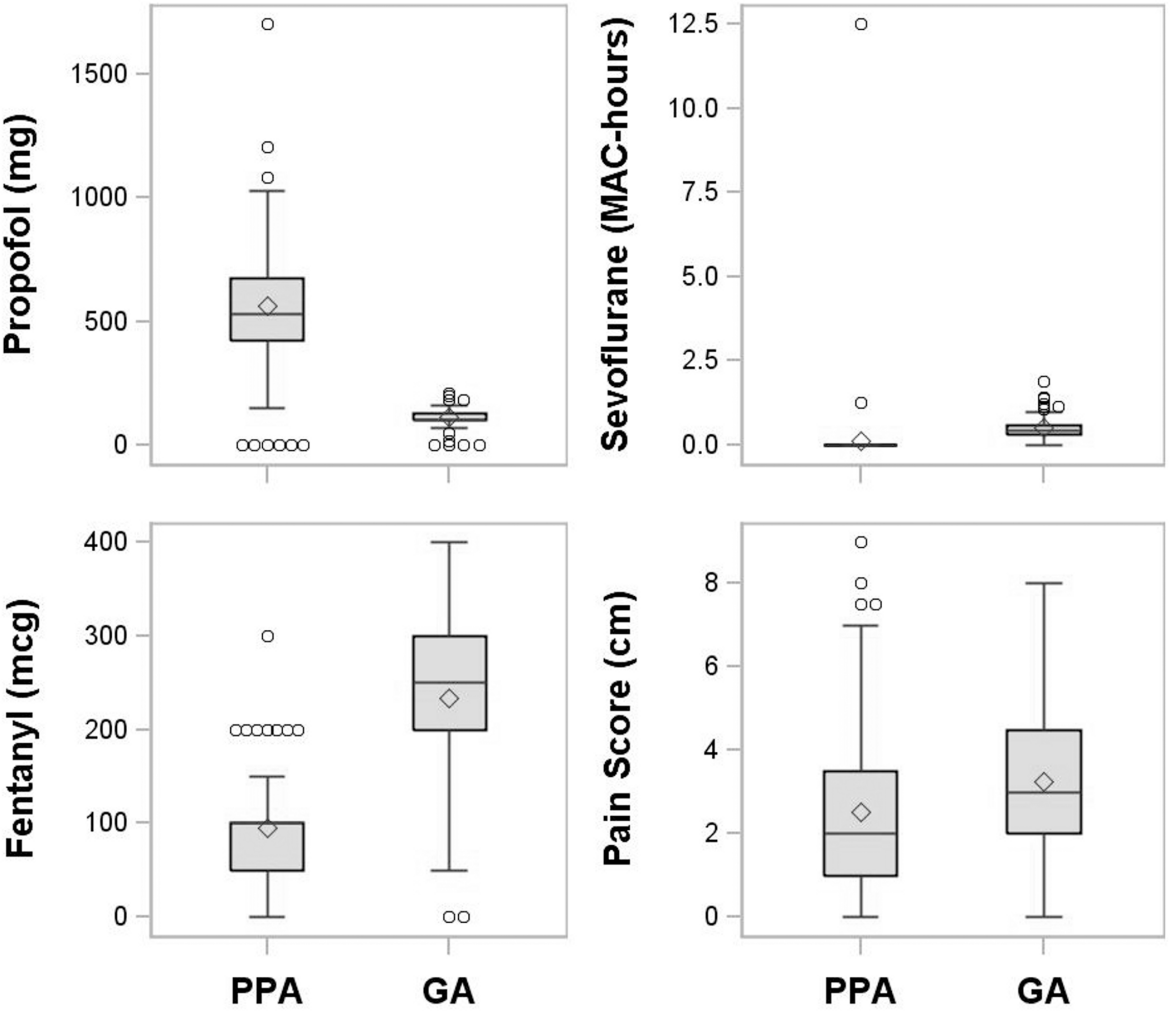
Boxplots comparing randomized groups for each primary outcome. The box represents the interquartile range, the horizontal line the median, the whiskers extend to the high and low values within 1.5 interquartile ranges of the box, the circles represent values beyond 1.5 interquartile ranges of the box and the diamond represents the mean.

**Fig 4:**
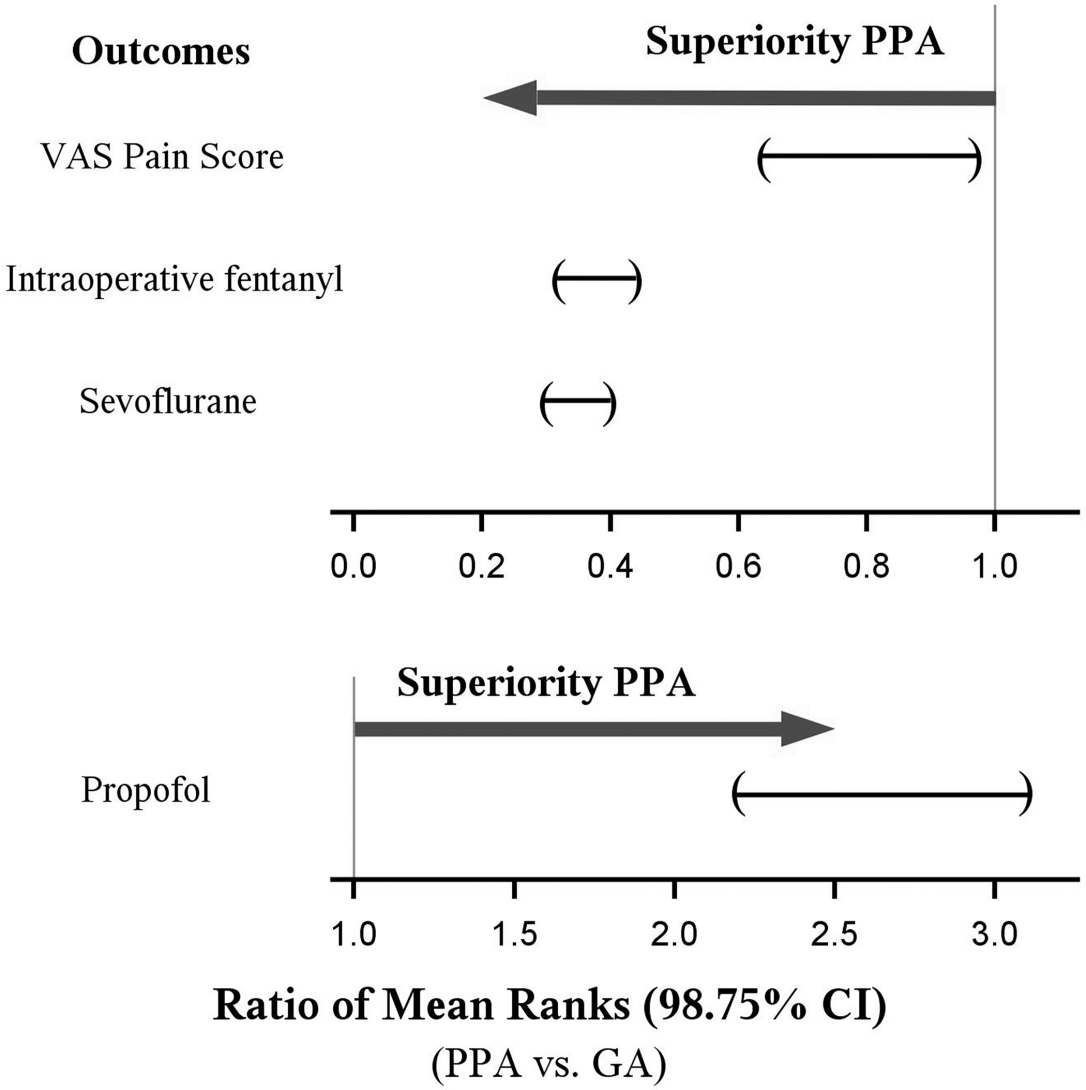
Superiority plots showing the ratios of mean ranks (98.75% confidence intervals, CIs) between the regional and general groups for each primary outcome. Parentheses indicate two-sided 98.75% CIs estimated using bootstrap resampling. Joint hypothesis testing of the four primary outcomes indicated effectiveness of paravertebral analgesia over general anesthesia, as all four CIs lie within the superiority regions: less pain, less intraoperative fentanyl use, less volatile anesthetic use, and increased propofol use.

Noninferiority on all four outcomes was found at the significant level of 0.025and superiority in the pre-specified direction was observed for all four outcomes (i.e., all *P* < 0.001, the same direction as the noninferiority tests). In one-tailed superiority tests, the PPA group had lower intraoperative sevoflurane and fentanyl consumption, lower postoperative pain scores, but higher propofol requirements than the GA group (Table 2, Fig 3). Furthermore, all four estimated 98.75% CIs fell within the superiority regions (Fig 4), indicating the effectiveness of PPA over GA as well. Results are presented as the median difference between the groups with 98.75% CIs, as well as the estimated ratio ofmean ranks and 98.75% CIs. The estimated ratio of fentanyl mean ranks of 0.38 (0.32, 0.44) indicated that the mean fentanyl dose was reduced (on the rank scale instead of the actual scale) by 62% (98.75% CI: 54% to 68%) compared with the GA group.

**Table 2:** Comparison of randomized groups (PPA vs. GA) on primary outcome variables (N = 247). Abbreviations: PPA, propofol and paravertebral block anesthesia; GA, general anesthesia; CI, confidence interval; MAC, minimum alveolar concentration. VAS = 10-cm visual analog scale. Morphine equivalents were calculated from long-acting opioids given intraoperatively or postoperatively, but excluded intraoperative fentanyl, which is reported separately. Results presented as median [first, third quartiles], median difference and ratio of mean ranks (98.75% confidence interval).

Outcomes	PPA group (N = 121)	GA group (N = 126)	Median Difference [98.75% CI]^	Ratio of Mean Ranks^#^ [98.75% CI]^	Non-inferiority P value*	Superiority P value*
**Propofol dose [mg]**	529 [424, 672]	100 [100, 130]	413(381, 454)	2.6 (2.2, 3.1)	<0.001	< 0.001
Sevoflurane consumption [MAC-hours]**	0 [0, 0]	0.4 [0.3, 0.6]	-0.4(-0.5, -0.4)	0.35 (0.30, 0.40)	<0.001	< 0.001
**Intraoperative fentanyl requirement [μg]**	100 [50, 100]	250 [200, 300]	-150(-150, -100)	0.38 (0.32, 0.44)	<0.001	< 0.001
**VAS for pain [cm]**	2.0 [1.0, 3.5]	3.0 [2.0, 4.5]	-1(-1.5, -0.01)	0.79 (0.64, 0.97)	<0.001	<0.001

^ 98.75% confidence intervals to maintain overall 0.025 significance level: one-tailed testing for superiority for four outcomes with one-tailed alpha of 0.00625 for each outcome.

* P-values from one-sided Wilcoxon rank sum test; Bonferroni multiplicity correction [i.e., significance criterion P <0.025/4 = 0.00625; thus, all are significant].

** Note: sevoflurane was administered to 2% of regional and 99% of general anesthesia patients (relative risk, 0.02; 98.75% CI, 0.001 to 0.1).

# Mean ranks: values for an outcome variable were first ordered from smallest to largest across all patients, and the ordering for a particular patient was considered as the “rank” for that outcome (i.e., there were a total of 121 + 126 = 247 ranks). The ranks were then averaged within each randomized group and the ratio of those means reported as “ratio of mean ranks”. Confidence intervals were obtained by bootstrap resampling.

We found that patients in the PPA group were more likely to have a higher heart rate, less likely to have a MAP<55mmHg,and less likely to complain of postoperative nausea or emesis (all *P* <0.05, Table 3), but there was no significant difference in intraoperative ephedrine use (*P* = 0.06).

**Table 3:** Secondary outcomes in the PPA and GA groups. Data are presented as mean ± standard deviation, median [first, third quartiles], or n (%). Abbreviations: PPA, propofol and paravertebral block anesthesia; GA, general anesthesia; CI, confidence interval; TWA, time-weighted average; MAP, mean arterial pressure.

Secondary outcomes	PPA group (N = 121)	GA group (N = 126)	Mean difference or relative risk (Regional–General) 95% CI	P Value**
**Intraoperative**				
**Ephedrine dose (mg)**	6 [0, 12]	9.5 [0, 12]	0 (-2, 0.01)*	0.06
**TWA MAP (mmHg)**	73.8 ± 7.6	74.1 ± 7.5	-0.27 (-2.2, 1.6)	0.78
** Any MAP >100**	14 (12)	19 (15)	0.77 (0.40, 1.46)	0.42
** Any MAP <70**	97 (80)	107 (85)	0.94 (0.84, 1.06)	0.32
** Any MAP <60**	34 (28)	49 (39)	0.72 (0.50, 1.04)	0.073
** Any MAP <55**	11 (9)	23 (18)	0.50 (0.25, 0.98)	0.037
**TWA heart rate (/min)**	75 ± 9	69 ± 8	5.8 (3.7, 7.9)	<0.001
**Postoperative**				
**Nausea (any time)**	48 (40)	75 (60)	0.67 (0.51, 0.87)	0.0018
** Recovery room**	16 (13)	33 (26)	0.50 (0.29, 0.87)	0.011
**Emesis (any time)**	27 (22)	51 (40)	0.55 (0.37, 0.82)	0.0021
**Recovery room**	4 (3)	16 (13)	0.26 (0.09, 0.76)	0.0068

* Median difference (95% CI).

** p values from t-test, Wilcoxon rank sum test or chi-square test, as appropriate.

No serious adverse events or deaths occurred in either group. Three patients in the PPA group developed a Horner’s syndrome soon after the block, but this had resolved by the first postoperative morning.

## Discussion

We observed a reduction in sevoflurane use by 65%, a reduction in fentanyl dose by 62%, and a concomitant increase in propofol use by up to 160% in patients anesthetized with propofol complemented by the TPVB regional technique. That paravertebral blocks are analgesic and reduce intraoperative opioid and volatile anesthetic requirement is unsurprising[1,12]. The reason why we chose volatile anesthetic dose, along with intraoperative fentanyl and propofol use, as primary outcomes to evaluate the efficacy of ultrasound-assisted multilevel TPVB, is the potential beneficial effect of regional analgesia on tumor recurrence and metastasis may result from degraded immune defenses in patients having cancer surgery under general anesthesia. Evidence shows that neutrophils isolated from patients undergoing surgery under spinal anesthesia exhibited superior chemotaxis than those from patients undergoing general anesthesia[19].

Other possible explanations include hemodynamic instability or compromise, the surgical stress response, poorly controlled postoperative pain and delayed recovery of spontaneous respiration. Many inflammatory mediators produced by leucocytes and endothelial cells will elicit pain, which can be counteracted by endogenous opioid peptides in the peripheral nerve terminals[20]. Inflammatory reactions arise in areas of trauma, leading to the activation of pain receptors[21]. Immuno-compromise may influence the risk of postoperative infection, the duration of wound healing, treatment response and tumor cell dissemination.

Surgical procedures and anesthetic techniques have been shown to inhibit the activity of NK and functional T-lymphocytes for several days[22–24]. Volatile anesthetics such as sevoflurane are reported to impair NK- and T-cell function [6,7], and acute and chronic administration of exogenous opioids inhibits components of the cellular and humoral immune responses, such as antibody production, NK cell activity, cytokine secretion, lymphocyte proliferative responses to mitogens and phagocytic activity[8].

In contrast, propofol appears to have little effect on the risk of metastasis, or may even be protective[9,10]. Propofol reported has anti-tumor effects by reducing the production of cyclooxygenase 2 and prostaglandin E2 by cancer cells[25].It is therefore possible that volatile anesthesia supplemented by opioids contributes to recurrence of cancer after potentially curative surgery[26], although this theory remains speculative. Furthermore, in the tumor microenvironment, the balance between T_helper1_ and T_helper2_cells is disturbed. Zhou and colleagues found that epidural anesthesia can reverse this immune imbalance more than general anesthesia, and potentially exerts a beneficial effect on patients with hepatocellular cancer by enhancing anti-tumor T_helper1_ polarization[27]. Suppression of humoral and cellular immunity by volatile anesthetics and opioids promotes angiogenesis and micro-metastasis. It is therefore logical to seek practical strategies to preserve immune function and improve clinical outcomes for cancer patients.

Analgesia in both our groups was clinically satisfactory, but patients given paravertebral analgesia had better outcomes, with less immediate postoperative pain, reflected by a reduction in VAS score of 21%.The intensity of pain in our study was broadly comparable with two randomized studies of paravertebral analgesia (with or without concomitant general anesthesia) versus general anesthesia alone [2,4].In an animal model, optimum postoperative analgesia independently reduced the metastatic burden in rats inoculated with breast adenocarcinoma cells following surgery[11], which informed our choice of the most intense pain in the immediate two postoperative hours as one of our primary outcome measures.

Real-time ultrasound guidance can be used to help identify the paravertebral space, guide needle placement and monitor the spread of local anesthetic. Multi-level block may be considered inefficient when compared with single-level puncture and insertion of a catheter to allow postoperative patient-controlled regional analgesia [28–31]. There is a risk of hematoma with the catheter technique that may rarely need surgical intervention [32].

In our healthcare system, the relatively high cost of the postoperative patient-controlled pump is not covered by basic medical insurance, hence our choice of a novel multi-level technique using ultrasound to identify the TP and pleura, combined with the traditional approach to perform TPVB. Using this technique, one clinician can perform TPVB in two or three patients in 15 minutes, and the ability to view the pleura reduces the incidence of complications. There were no serious adverse events or deaths in either group; the incidence of Horner’s syndrome in the PPA group was similar to that in previous studies [33].

The combination of regional anesthesia and general anesthesia may result in significant hemodynamic fluctuations [34], potentially causing serious adverse events such as asystole [35]. The TPVB can be thought of as a unilateral thoracic epidural block, and there are few if any clinically significant hemodynamic effects in patients following mastectomy. Our results showed that patients in the PPA group were more likely to have a higher heart rate, but less likely to have a MAP <55 mmHg, although there was no significant difference in intraoperative ephedrine use between the groups. Postoperative nausea and vomiting continues to be one of the most common postsurgical medical problems [36,37]: regional anesthesia followed by regional analgesia may reduce its incidence [6–10]. Our results were generally consistent with previous reports, and showed that patients given paravertebral analgesia had less postoperative nausea and emesis both in the PACU and at any time during the postoperative period.

## Conclusions

In summary, our findings were largely consistent with previous studies. The combination of propofol with ultrasound-assisted TPVB reduces intraoperative volatile anesthetic and opioid requirements, and the intensity of postoperative pain, in patients undergoing breast cancer surgery, but requires more propofol to be administered. Whether regional analgesia reduced cancer recurrence remains to be determined in large randomized outcome trials.

## Supporting Information

S1 CONSORT Checklist CONSORT Checklist. (DOC)

S1 Protocol Trial Protocol. (PDF)
