# Supplementary material for: Ultrasound-Assisted Thoracic Paravertebral Block Reduces Intraoperative Opioid Requirement and Improves Analgesia after Breast Cancer Surgery: A Randomized, Controlled, Single-Center Trial
Source: PLoS One. 2015 Nov 20;10(11):e0142249. doi: 10.1371/journal.pone.0142249 (PMC4654480; doi:10.1371/journal.pone.0142249)
Supplement: S1 Protocol — (PDF) [file pone.0142249.s002.pdf]

Regional Anesthesia and Breast Cancer Recurrence

Daniel I. Sessler, M.D. and Colleagues

Department of OUTCOMES RESEARCH

The Cleveland Clinic
9500 Euclid Ave
Cleveland, OH 44195

Tel: 216-444-4900
Fax: 216-444-61353
Email: DS@OR.org
Web: www.OR.org

Abstract

Surgery is the primary and most effective treatment of breast cancer, but residual disease in the form of scattered micrometastases and tumor cells are usually unavoidable. Whether minimal residual disease results in clinical metastases is a function of host defense and tumor survival and growth. At least three perioperative factors shift the balance toward progression of minimal residual disease: (1) Surgery *per se* depresses cell-mediated immunity, reduces concentrations of tumor-related anti-angiogenic factors (*e.g.*, angiostatin and endostatin), increases concentrations of pro-angiogenic factors such as VEGF, and releases growth factors that promote local and distant growth of malignant tissue. (2) Anesthesia impairs numerous immune functions, including those of neutrophils, macrophages, dendritic cells, T-cell, and natural killer cells. (3) Opioid analgesics inhibit both cellular and humoral immune function in humans, increase angiogenesis, and promote breast tumor growth in rodents. However, regional analgesia attenuates or prevents each of these adverse effects by largely preventing the neuroendocrine surgical stress response, eliminating or reducing the need for general anesthesia, and minimizing opioid requirement. Animal studies indicate that regional anesthesia and optimum postoperative analgesia independently reduce the metastatic burden in animals inoculated with breast adenocarcinoma cells following surgery. Preliminary data in cancer patients are also consistent: paravertebral analgesia for breast cancer surgery reduced risk of recurrence or metastasis approximately four-fold (95% CI of estimated hazard ratio is 0.71 - 0.06) during a 2.5 to 4-year follow-up period compared to opioid analgesia. Similar results were observed with epidural analgesia for prostate surgery. We will thus test the hypothesis that recurrence after breast cancer surgery is lower in patients randomized to regional anesthesia & analgesia with propofol sedation than to sevoflurane general anesthesia and opioid analgesia. In a Phase III, multi-center trial, Stage 1-3 patients having mastectomies will be randomly assigned to paravertebral or thoracic epidural anesthesia/analgesia, or to general anesthesia and morphine analgesia. Enrolling 1,100 patients over 5 years will provide an 85% power for detecting a 30% treatment effect at an alpha of 0.05. Confirming our hypothesis will indicate that a small modification to anesthetic management, one that can be implemented with little risk or cost, will reduce the risk of cancer recurrence — a complication that is often ultimately lethal.

Version 89
03/02/2011

Personnel

Key Personnel

Name	Organization	Role on Project
Daniel I. Sessler, M.D.	CCLCM-CWRU*	Principal investigator
Andrea Kurz, M.D.	Hillcrest Hospital	Site director, Cleveland Clinic Main Campus
Kennith Cummings III, M.D.	CCLCM-CWRU	Site director, Cleveland Clinic Hillcrest
Sabry Ayad, M.D.	Fairview Hospital	Site director, Cleveland Clinic Fairview
Donal Buggy, M.D.	Mater Misericordia Hospital	Site director, Dublin
Alparslan Turan, M.D.	University of Louisville	Site director, Louisville
Edith Fleischmann, M.D.	University of Vienna	Site director, Vienna
Adrian Alvarez, M.D.	Hospital Italiano	Site director, Buenos Aires
Sven-Egron Thorn	Orebrto Hospital	Site director, Orebro
Tanja A. Meyer-Treschan	University of Düsseldorf	Site director, Düsseldorf
Edward Mascha, Ph.D.	CCLCM-CWRU	Principal statistician

*Cleveland Clinic Lerner College of Medicine, Case Western Reserve University.

Consultants and Collaborators

Name	Organization	Role on Project
Michael Ritchey, M.D.	CCLCM-CWRU	Collaborator, anesthesiologist
Roseann Petcharka	Hillcrest Hospital	Research coordinator
Joseph Crowe, M.D.	CCLCM-CWRU	Co-investigator, breast surgeon
Thomas Budd, M.D.	CCLMC-CWRU	Co-investigator, oncologist
Shamgar Ben-Eliyahu, Ph.D.	Tel Aviv University	Co-investigator: immune effects of anesthetics, opioids and surgery
Jim Malgieri, M.D.	CCLCM-CWRU	Consultant, breast surgeon

A. Specific Aims

"It may seem strange that a man of science should believe a thing of this kind — an idle tale for the ignorant and superstitious, you will say — but I do believe it. And if you would know why, listen:" (W.H.C. Pynchon)

Breast cancer is the most common cancer in women and the second leading cause of cancer death. Treatment hinges on effective surgical removal of the primary tumor, but recurrence occurs in a significant portion of patients. **Even with the best technique, tumor surgery is usually associated with release of tumor cells into the lymphatic and blood streams and a large fraction of patients already harbor micrometastases and scattered tumor cells at the time of surgery.**[1-3]

Whether this *minimal residual disease* results in clinical metastases depends largely on the balance between anti-metastatic immune activity and the tumor's ability to seed, proliferate, and attract new blood vessels.[4-6] In practice, the immune system and other host defenses frequently fail to neutralize minimal residual disease; consequently, local recurrence and metastatic disease remains common after breast cancer surgery. At least three perioperative factors shift the balance toward progression of minimal residual disease:

- The first is surgery *per se,* which releases tumor cells into circulation,[1-3] depresses cell-mediated immunity including cytotoxic T-cell and natural killer (NK) cell functions,[7-10] reduces circulating concentrations of tumor-related anti-angiogenic factors (*e.g.*, angiostatin and endostatin),[11-14] increases concentrations of pro-angiogenic factors such as VEGF,[15,16] and releases growth factors that promote local and distant growth of malignant tissue.[5]
- The second factor is that anesthesia, *per se*, which impairs neutrophil, macrophage, dendritic-cell, T-cell, and NK-cell immune functions.[17-20]
- The third is opioids, which are given to control surgical pain. Opioids inhibit both cellular and humoral immune function in humans.[17,21,22] Furthermore, morphine is pro-angiogenic and promotes breast tumor growth in rodents.[23] Consequently, non-opioid analgesia helps preserve natural killer cell function in animals and humans and reduces metastatic spread of cancer in rodents.[8]

Regional *anesthesia* (intra-operative blockade of nociception) and *analgesia* (postoperative pain relief) attenuate or prevent each of these adverse effects. For example, regional anesthesia largely prevents the neuroendocrine stress response to surgery by blocking afferent neural transmission from reaching the central nervous system and activating the stress response, and by blocking descending efferent activation of the sympathetic nervous system.[24-26] As might thus be expected, surgical stress is attenuated better by regional than by general anesthesia. Consequently, NK-cell function is better preserved and metastatic load to the lungs is reduced in a rat model of breast cancer metastasis.[7]

When regional and general anesthesia are combined, the amount of general anesthetic required is much reduced — as is, presumably, immune suppression. Furthermore, regional analgesia provides superb pain relief, essentially obviating the need for postoperative opioids, and the consequent adverse effects on immune function and of tumor growth.[17,22,26] Regional analgesia also reduces release of endogenous opioids.[27]

Available data thus suggest that regional anesthesia and analgesia help preserve effective defenses against tumor progression by attenuating the surgical stress response, by reducing general anesthesia requirements, and by sparing postoperative opioids. Animal studies are consistent with this theory, showing that regional anesthesia and optimum postoperative analgesia independently reduce the metastatic burden in animals inoculated with breast adenocarcinoma cells.[7,9] Our preliminary data in cancer patients are also consistent with this theory: paravertebral anesthesia and analgesia for breast cancer surgery was associated with an approximately four-fold reduced risk of recurrence or metastasis during a 2.5 to 4-year follow-up period (95% CI of estimated hazard ratio is 0.71 - 0.06).

We propose a Phase III, multi-center study of patients having mastectomies for breast cancer. Patients will be randomly assigned to 1) paravertebral or thoracic epidural anesthesia/analgesia[28-31] or 2) general anesthesia combined with postoperative patient-controlled morphine analgesia. Specifically, we will test the primary hypothesis that the recurrence of local and metastatic cancer after primary breast cancer surgery is reduced when patients receive intraoperative and postoperative regional anesthesia and analgesia rather than

general anesthesia and opioid analgesia. A total of 1,100 patients enrolled over 5 years will provide a 85% power to detect a 30% treatment effect at an alpha value of 0.05.

The significance of our proposed study is that it addresses a major women's health problem (cancer recurrence) and suggests an intervention that is safe, inexpensive, and easy to implement.[32] The solution we propose is innovative in challenging the current practice of using mostly general anesthesia and opioid analgesia for cancer surgery. Our approach will be to conduct a large, multi-center randomized trial that will provide "gold-standard" evidence to support or refute our hypothesis. All participating investigators have considerable experience with clinical research in general, and specifically with the proposed methodologies. The study sites will all be OUTCOMES RESEARCH affiliates, each of which has substantial experience with large outcome trials; the overall environment for this trial is thus excellent.

B. Background and Significance

There are 211,000 new cases of invasive breast cancer diagnosed each year in the United States, and the disease causes approximately 40,000 deaths annually. One woman in eight develops breast cancer, and breast cancer is the most common type of cancer and the second most common cause of cancer death in women.[33] Treatment hinges on effective surgical removal of the primary tumor. Failure of effective treatment is marked by local recurrence or metastatic disease — with either often being an ultimately lethal event.

In this section, we will start by reviewing the basic physiology of tumor metastasis, including recent information about the importance of angiogenic factors for tumor development and promotion of angiogenesis by surgery. We will then discuss the role of the immune system in controlling minimal residual disease after the removal of the primary tumor, the immunosuppressive effects of surgery *per se*, the immunosuppressive effects of general anesthetics and opioids, the promotion of tumor-induced angiogenic factors by opioids, and finally the mechanisms by which regional anesthesia and analgesia might ameliorate each of these metastasis-enhancing effects.

Tumor Recurrence and Metastasis: Angiogenesis and Cell-mediated Immunity

Tumor cells are able to spread from a primary neoplasm and colonize other organs, a phenomenon named metastasis. Tumor metastasis is the most common cause of death in cancer patients.[34] Malignant cells are probably released nearly continuously from most solid tumors in humans. This highly selective and sequential process involves tumor cell invasion of the surrounding tissue, evasion of host defense, penetration into the lymphatic system or blood stream, circulation, arrest in organs, adherence to vessel wall, extravasation, and proliferation at secondary sites where, after vascularization, the cancer cells can form life threatening tumors. Only tumor cells that can succeed in all these steps have the capacity to metastasize. The molecular mechanisms enabling this sequence of events are still being uncovered;[38] however, tumor angiogenesis is known to be a critical component of metastasis. Primary tumors that are highly vascularized provide increased contact between tumor cells and the circulation; consequently, barrier function is reduced because tumor angiogenesis results in immature, highly permeable blood vessels that facilitate transmigration of tumor cells into the circulation.[37]

Judah Folkman's group has demonstrated that angiogenesis inhibitors released into the circulation by or in consequence of the primary tumor can inhibit metastatic growth, and that removal of the primary tumor enables vascularization and growth of pre-existing micrometastases.[39,40] More recently, a mechanism has been established whereby hematopoietic progenitors localize to tumor-specific premetastatic sites where they create a microenvironment conductive to future incoming tumor cells.[38]

The suggestion that protecting cell-mediated immunity from postoperative suppression may reduce long-term recurrence rates critically hinges on the assumption that cell-mediated immunity can control minimal residual disease after the removal of the primary tumor. Although animal studies have repeatedly supported this suggestion, these studies were justifiably criticized for not simulating the natural course of primary tumor development and the metastatic process in humans.[41,42] Clearly, in patients presenting with a primary tumor, the immune system has failed to control the major bulk of the malignant tissue.

Nonetheless, several lines of evidence in humans suggest that cell-mediated immunity limits the metastatic process and that the removal of the primary tumor presents cell-mediated immunity with an opportunity to eliminate or control minimal residual disease. For example, NK activity and the response of

mixed lymphocytes toward autologous malignant cells predict long-term survival in humans better than stage and grade of tumors.[43,44] Additionally, even though most cancer patients harbor minimal residual disease after surgery, only a portion of them subsequently develop metastases.[45] Lastly, various surgical procedures shown to improve or worsen postoperative levels of cell-mediated immunity affect recurrence rates. We have recently reviewed in detail the evidence from human and animal studies supporting a critical role for cell-mediated immunity in controlling metastasis following the removal of a primary tumor.[5]

Cancer Surgery Facilitates Metastases: A Decisive Period for Intervention

Surgical removal of the primary breast tumors remains a major modality of cancer therapy, eliminating the main pool of metastasizing cells. Chemotherapy, endocrine therapy, and radiotherapy all have significant roles, but surgical removal of the tumor usually offers the best prospect for an improved prognosis.[46] Surgical treatment of a primary tumor rarely removes all malignant foci, especially pre-existing scattered micrometastases. It does, though, remove the major tumor bulk of metastasizing cells, providing host defenses an opportunity to eradicate residual disease. However, several mechanisms exist by which surgery *per se* might accelerate the metastatic process:

- Tumor cells are released into the circulation by the unavoidable physical manipulation of the tumor or its vascularization in humans.[1,47,48] Surgery facilitates metastasis by suppressing tumor-directed immunity,[49,50] impairing the detection and destruction of circulating tumor cells, and upsetting the balance between proliferation and tumoricidal activity within established metastases[51];
- Removal of the tumor decreases the concentration of anti-angiogenic factors such as angiostatin and endostatin,[11,14,39,40] which are metabolites or degradation products released from the host extracellular matrix in response to damage by tumor invasion.
- Stress hormones released postoperatively, including catecholamines, promote release of pro-angiogenic factors such as VEGF[15,16];
- Growth factors, which are released by injured tissue to facilitate healing, also promote local and distant growth of malignant tissue.[52,53]

Fig.1. Multiple factors associated with cancer surgery simultaneously increase the risk of metastasis. The perioperative period represents a *decisive period* and an opportunity to eliminate or control minimal residual disease.

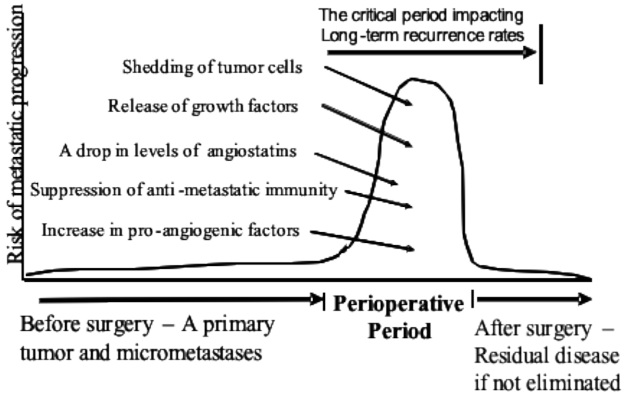

These five risk factors occur simultaneously during and immediately after surgery, consequently activating dormant micrometastases and establishing new metastases. During and immediately after surgery is a *decisive period* during which interventions that promote effective host defense may be especially beneficial (Fig. 1). As developed below, anesthetic and analgesic techniques have a significant impact on these processes, providing an opportunity to reduce the risk of metastases.

Surgical Stress is Immunosuppressive

The inflammatory and neuroendocrine responses to surgical tissue injury eventually lead to compromised immunocompetence in both humans and animals.[54] After major surgery, there is a sharp increase in plasma concentrations of acute inflammatory cytokines (IL-6 and IL-8), prostaglandins (PG-E2), and stress hormones (catecholamines, corticosteroids); and a decrease in Th1 cytokines. These alterations profoundly suppress cell-mediated immunity in animals and humans.[5,55-58] Cytokine balance, circulating

effector cell numbers, and *ex-vivo* effector cell function are all altered in animals and humans.[18,59] A relevant example is natural killer (NK) cell activity.

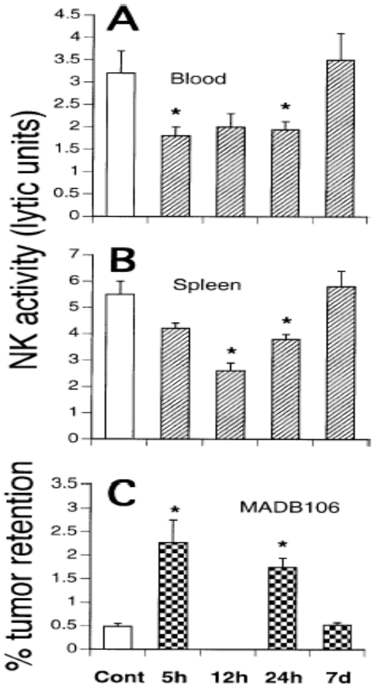

Fig. 2. Time course of the effects of surgery on NK activity in blood *(A)* and spleen *(B)* (both in lytic units) and on MADB106 lung tumor retention *(C), an index of the metastatic process*. Rats served as controls (Cont) or underwent surgery at either 5, 12 or 24 hr, or 7 days before blood and spleen were taken, or at either 5 or 24 hr, or 7 days before MADB106 tumor cells were inoculated for the assessment of 24-hr lung tumor retention (rats were not inoculated with the tumor at 12 hr after surgery because the 24-hr period starting at this point is covered by the 5- and 24-hr groups). Significant suppression of blood and splenic NK activity was evident up to 24 hr post-surgery but not at 7 days. Correspondingly, a significant increase in tumor retention was evident if tumor cells were injected up to 24 hr post-surgery but not at 7 days. Error bars represent SEM. *Significant difference from the respective control group. From Ben-Eliyahu, S., et al. Evidence that stress and surgical interventions promote tumor development by suppressing natural killer cell activity. Int J Cancer 80:880-8, 2002.

NK-cells are a subpopulation of lymphoid cells that spontaneously recognize and kill a variety of tumor cells *in vitro* and *in vivo*[60] and are known to play a determinant role in controlling tumor development — and especially the metastatic process.[61] **Suppression of NK-cell activity occurs within hours of surgery, lasts for few days, and is proportional to the invasiveness of the surgery**.[18,62] Tissue damage, inflammation, pain, anesthetic and analgesic compounds, and psychological stress all contribute to NK-cell suppression and the tumor-promoting effects of surgery (Fig. 2).[63-66] Major surgery in humans and in animals also suppresses other important aspects of innate immunity including cytotoxic T lymphocytes (CTLs), macrophages, and dendritic cells.[67-69]

In addition to the surgery *per se*, several factors associated with surgery impair perioperative immune competence. Among these are hemorrhage,[70] which causes immunosuppression correlated with the volume of lost blood,[5] and allogenic blood transfusion, which reduces NK activity[71] and increases cancer recurrence in humans after operations designed to be curative.[72] Hypothermia suppresses lymphocyte proliferation, macrophage phagocytosis, and NK activity, and also exacerbates the immunosuppressive effects of surgery.[5] However, hypothermia *per se* does not increase the recurrence of colon cancer.[73] Highly relevant to breast cancer surgery is the fact that estrogens modulate stress-induced immunosuppression.[51,74] Pain suppresses NK-cell function and promotes tumor metastasis,[75] and the opioid antagonist naltrexone prevents the immunosuppressive effects of surgery.[76] Stress — including stressors far less intense than surgery — also impairs NK-cell function, an effect that is prevented by adrenergic blockers.[77,78]

General Anesthetics are Immunosuppressive and Tumor-promoting

Ketamine, thiopental, and halothane each have been shown to suppress NK-cell activity and promote metastasis in an animal model (Fig. 3).[79] Other volatile anesthetics also impair NK-cell function[18] by as much as 90%.[19] Halothane and isoflurane comparably reduce neutrophil motility[80] and sevoflurane impairs T lymphocytes.[81] While the immune effects of other volatile anesthetics differ somwhat,[82,83] most — including the newer agents sevoflurane and desflurane — appear to substantially inhibit various immune functions. It is likely that anesthetic-induced impairment of NK-cell activity is most pronounced in the context of the stress response to surgery.[84]

Nitrous oxide depresses chemotactic migration by monocytes, apparently by interfering with microtubules.[85] There is also *in vitro* evidence indicating that exposure to nitrous oxide inactivates vitamin B_{12}

and as a result methionine synthase.[86] Methionine synthase is the enzyme responsible for both conversion of homocysteine to methionine and methyltetrahydrofolate to tetrahydrofolate. Both are critical pathways for thymidine formation; thymidine is essential in DNA formation. Even after brief periods of nitrous oxide administration, DNA synthesis remains abnormal until the fourth postoperative day and does not return to normal until the sixth day.[87] This restricts formation of new cells, including the immune cells critical for fighting tumors. Inhibition of methionine synthase may explain the link between nitrous oxide exposure and spontaneous abortion.[88] By the same mechanism, nitrous oxide might impair proliferation of cancer cells. There is currently no evidence that nitrous oxide is better or worse than volatile anesthetics in terms of promoting metastases.

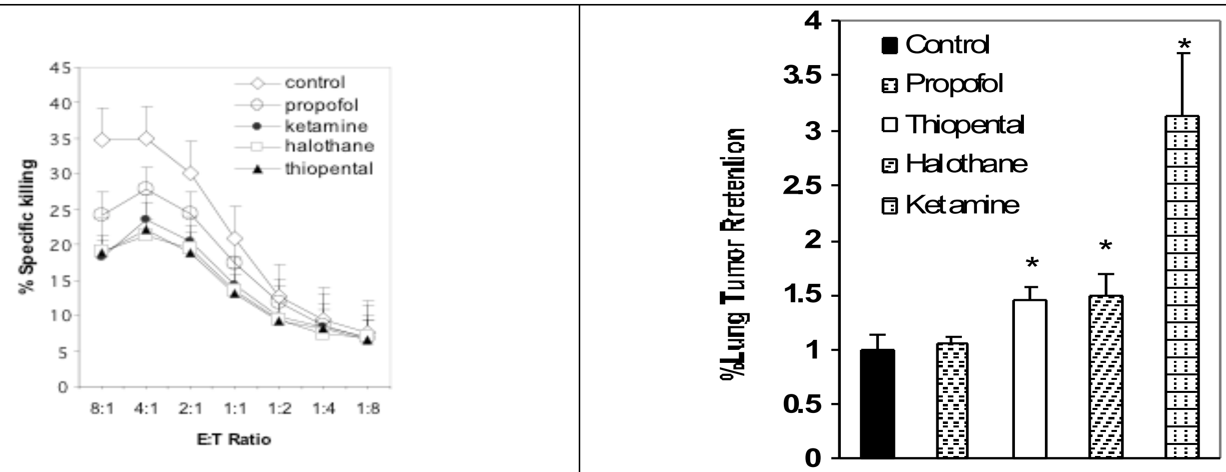

Fig. 3. Rats were anesthetized for 1 hour with halothane (2-3%) or infusion of ketamine (74 mg kg^{-1} hr^{-1}), propofol (83.3 mg kg^{-1} hr^{-1}), or thiopental (92.5 mg kg^{-1} hr^{-1}). Activity of NK-cells was assessed 3.5 h after induction of anesthesia. A parallel group of animals was simultaneously inoculated I.V. with radiolabeled MADB106 breast tumor cells, and lung tumor retention (LTR) assessed at 24 h. Mean ± S.E.M; * indicates a significant difference from control. **(Left)** Percent specific killing per ml blood to different effectors: target (ET) ratios (NK:YAC-1). Ketamine, halothane, and thiopental, but not propofol, significantly suppressed NK activity. **(Right)** Percent of LTR of radiolabeled MADB106 tumor cells. Ketamine, halothane, and thiopental, but not propofol treated rats had significantly higher LTR compared to controls.[79]

Propofol, ketamine, and midazolam markedly increase the release of inflammatory cytokines in human cells.[57,58] Propofol impairs neutrophil phagocytosis and killing of bacteria in humans,[89] and propofol and thiopental comparably impair neutrophil polarization.[90] Propofol also decreased pokeweed mitogen-induced lymphocytic responses in one study,[91] but had no effect in another.[56] The effects of propofol and isoflurane were compared with respect to a variety of immune responses including total leukocytes, percentages of lymphocyte subpopulations (CD3, CD4, CD8, CD20, CD16), and monocytes (CD14). Additionally, phytohemagglutinin-, concanavalin A- and pokeweed mitogen-induced and unstimulated lymphocyte proliferative responses, polyclonal immunoglobulin synthesis, and serum cortisol concentrations were explored. The immune response to ophthalmic surgery was basically similar in both anesthetic groups.[92]

In contrast, propofol appears to have no effect or possibly even protects against metastases (as also seen in Fig. 3, Right): clinically relevant concentrations of propofol (1-5 µ/ml) decrease the invasion ability of human cancer cells *in vitro*. In HeLa cells treated with propofol, focal adhesion and the formation of actin stress fibers were inhibited while propofol had little effect on the invasion ability of the HeLa cells with active Rho A. Furthermore, continuous propofol administration either had no effect on metastasis of a breast cancer cell line in rats[79] or inhibited pulmonary metastasis of murine osteosarcoma cells in mice.[93] In contrast, halothane, ketamine, and thiopental all increase metastases in similar studies.[79]

Opioids and Metastasis

The metastatic process is selective for cancer cells that succeed in *all* steps of the process detailed previously.[36] Any factor facilitating one or more of these steps will presumably augment the probability of metastasis. Considerable evidence suggests that opioids facilitate metastasis both *via* suppression of critical anti-tumor immune functions and by mechanisms independent of immune suppression.

Acute and chronic administration of exogenous opioids inhibits components of the cellular and humoral immune function such as antibody production, NK-cell activity, cytokine secretion, lymphocyte proliferative responses to mitogens, and phagocytic activity.[94,95] The immunosuppressive effects of morphine are best studied;[22,96] however, other opioids, including fentanyl[21,97] and subtype-specific opioid receptor agonists (Fig. 4),[98] produce comparable immune suppression in most studies.[68] Inhibition appears to be dose-dependent.[21]

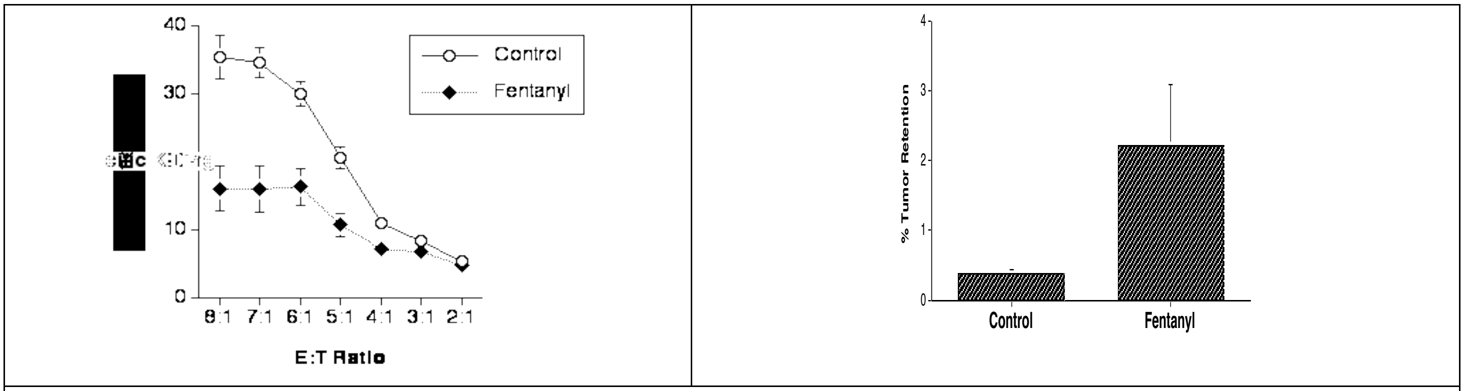

Fig. 4. Rats were injected with fentanyl (0.15 mg/kg). One hour later, specific killing was assessed at different Effector:Target ratios (NK:YAC-1). Fentanyl significantly suppressed NK cytotoxicity compared with control values (left). A parallel group of rats were injected IV with radiolabeled MADB106 tumor cells at the same time; fentanyl significantly increased tumor retention (by 5.8-fold) compared to controls (right).[97]

Opioids are now known to modulate immune response by a combination of central and peripheral mechanisms.[99] Endogenous and exogenous opioids bind three major types of receptors: the mu-, delta-, and kappa-opioid receptors that have been identified not only in peripheral sensory neurons and the CNS, but also in cells of the immune system such as PMNs, macrophages, T-lymphocytes, splenocytes, and macrophage-like and T-cell-like cell lines.[95] Opioids also alter the macrophage protein expression profile[100] and impair macrophage function including chemotaxis and phagocytosis. And finally, opioids reduce B-cell proliferation and antibody production.[101] In contrast, a number of studies have failed to find a direct effect of morphine on NK-cell activity *in vitro*,[101] suggesting CNS-mediated effects.

Signals of central origin may be relayed through 1) the hypothalamic-pituitary-adrenal axis resulting in the production of glucocorticoids, which are immunosuppressive, and 2) the sympathetic nervous system eliciting the release of biologic amines into lymphoid organs, which, in turn, also reduce immuno-competence.[102] Rahim et al. thus proposed that the HPA axis is involved in the immunosuppression by chronic opioid exposure, while the sympathetic nervous system mediates the immunosuppression induced by acute opioid administration.[103]

The extent to which opioid-induced immune suppression is central or peripheral in origin remains unclear.[99] Certainly, central opioid receptors can cause immune suppression.[104] For example, Hernandez found that systemic injection of a morphine analog that does not cross the blood-brain barrier did not impair immune responses, whereas small doses injected centrally did.[105] Similarly, injection of opioids into the periaqueductal gray matter suppressed NK-cell activity, whereas injection in other areas did not. However, intracerebral injections are quite different from spinal or epidural injections: Hamra compared equi-analgesic doses of subcutaneous and intrathecal morphine, and found that only the subcutaneous dose impaired cell-mediated immunity;[106] It is thus unlikely that the tiny opioid doses that are sometimes used clinically in conjunction with local anesthetics for spinal or epidural blocks produce substantial immune inhibition.

Opioids Upregulate Nitric Oxide Production and Angiogenesis

In vitro exposure of human leukocytes to morphine upregulates constitutive endothelial and neuronal nitric oxide synthases.[100] Furthermore, human endothelial cells express opioid receptor(s) and respond to opiates by increasing intracellular calcium concentration and nitric oxide (NO) production.[107,108] Morphine-induced increases in NO induce vasodilation in aortic rings *ex vivo*. NO also mediates increased vascular permeability, which could both increase release of cancer cells into the circulation before tumor excision and, subsequently, increase extravasation of circulating tumor cells and the formation of new metastases.

Nitric oxide also regulates endothelial cell proliferation, migration, and protease release — all of which are important for angiogenesis. Endothelial NO synthase (eNOS) plays a central role in endothelial cell migration and angiogenesis.[109] Morphine increases angiogenesis as evidenced by enhanced endothelial cell proliferation and tube formation *in vitro*, and increases *in vivo* blood-vessel formation in a matrigel plug assay and in breast-tumor xenographs.[23] And although one study showed reduced angiogenesis in the chicken chorioallantoic assay in the presence of morphine,[110] more recent work suggests that morphine and other opioids improve wound healing *via* an angiogenesis mechanism.[111]

Vascular Endothelial Growth Factor (VEGF) plays a key role in angiogenesis, prompting researchers to investigate whether surgery and/or surgery-related factors could affect the circulating concentrations of VEGF. In cultured endothelial cells, morphine inhibits the hypoxia-induced expression of VEGF,[112] but the effect of morphine on normoxic endothelial cells was not tested. In a recent study, patients having surgery for breast cancer under general anesthesia were given either paravertebral or opioid analgesia and tested for serum VEGF concentrations perioperatively; concentrations in the two groups were similar.[26] However, circulating VEGF *receptor* levels were not measured in that study. This is a critical limitation since circulating soluble forms of VEGF receptors act as negative regulators of angiogenesis because they bind to VEGF and form inactive complexes.[113-115] Concentrations of VEGF detected in serum, therefore, do not directly reflect the activity of this growth factor. For example, in breast-cancer patients the concentrations of VEGF are higher and the concentration of soluble VEGF-receptor-1 lower than in healthy controls; statistical significance of the difference between control and patients was therefore increased considerably when the results were expressed as the ratio of VEGF-receptor to VEGF rather than VEGF concentration *per se*.[116] More recent evidence indicates that opioids promote VEGF-induced angiogenesis, an effect that is nearly completely blocked by opioid antagonists.[117]

Regional Anesthesia Blocks Surgical Stress and Reduces Both General Anesthetic and Opioid Requirements

The severity of perioperative immunosuppression has prompted researchers and clinicians to suggest various strategies aimed at reducing consequences that are likely to be particularly deleterious in cancer surgery. These strategies include minimally invasive surgery to reduce tissue damage,[118] pharmacological and immunological interventions targeting the neuroendocrine and cytokine responses that mediate immune suppression,[119] development of novel synthetic opioids that retain analgesic properties but lack immunosuppressive effects,[120] and use of peripheral opioid antagonists that may prevent the peripheral immunosuppressive effects of opioids while retaining their central analgesic properties.[99,121] Another major strategy to attenuate the multifactorial immunosuppressive and tumor-promoting effects of cancer resection is the use of regional anesthesia and analgesia.

Inflammatory markers and immune function are similar with general and regional anesthesia in non-surgical volunteers who do not have concomitant tissue injury.[122,123] However, regional anesthesia markedly attenuates the neuroendocrine response to surgery, as evidenced by smaller perioperative concentrations of stress-induced plasma catecholamines and cortisol in patients receiving regional anesthesia compared to those given general anesthesia.[26,124] These alterations were expected to reduce postoperative immune suppression, as were indeed reported. For example, intraoperative neutrophil function is better preserved during spinal anesthesia than during either halothane or isoflurane anesthesia in rabbits.[80] Similarly, NK and Th1 cell activity is better preserved when epidural anesthesia is used compared with general anesthesia in humans.[125-128]

The consequences of these clinical observations have been evaluated in an animal model.[7] Rats were subjected to halothane anesthesia alone, with systemic morphine, or with spinal block. They underwent laparotomy or were left undisturbed, and were inoculated with metastatic adenocarcinoma cells to mimic the cancer cell shedding that occurs during curative resection. Laparotomy under halothane anesthesia alone increased lung retention of tumor cells up to 17-fold. The addition of spinal blockade reduced this effect by 70%. A similar pattern was observed when studying the actual development of metastasis (Fig. 5). Findings were nearly identical in a more recent study in mice.[129] There is thus compelling small-animal evidence that regional analgesia reduces metastasis of experimental cancer.

Fig. 5. The effect of surgery on the number of pulmonary metastases (mean + SEM). In rats undergoing general (halothane) anesthesia (G.A.), **surgery** significantly increased the number of pulmonary metastases compared to the non-operated **control** group. Adding spinal block (G.A. + spinal block) abolished this increase. Asterisk denotes statistically significant differences. Reproduced from data presented in: Bar-Yosef.[7]

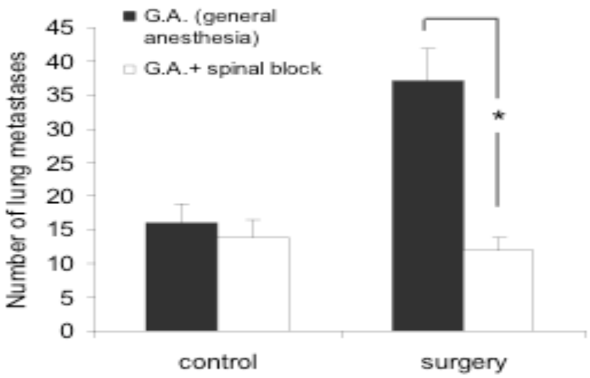

In addition to attenuating the stress response, regional analgesia obviates the need for postoperative opioids by producing profound analgesia.[32] In patients receiving regional analgesia versus intravenous morphine to alleviate postoperative pain, the neuroendocrine response is attenuated and the NK-cell activity preserved.[125,130] Regional analgesia also reduces release of endogenous opioids.[27] Consistent with this theory is evidence that melanoma recurrence rates in patients are 40% higher with general anesthesia than with local anesthesia, even after multivariate compensation for other prognostic factors.[131]

Regional Anesthesia and Chronic Pain

Chronic postsurgical pain is defined as pain developing after a surgical procedure that lasts at least three months for which other causes (i.e., malignancy or chronic infection) have been excluded.[132] Chronic postsurgical pain reportedly occurs in 31-49% of patients after breast surgery.[133] Other studies suggest that the incidence of chronic pain after thoracotomy may be as high as 50% one year after surgery.[134] Some level of residual pain is reported for up to several months after the procedure in about half of patients undergoing lower abdominal surgery.[135-137] Approximately 25% of patients report pain 1 yr after sternotomy[138,139] or herniorrhapy.[138,140]

The causes of chronic postoperative pain remain obscure. However, excellent acute analgesia may protective. Patients report that acute postoperative analgesia is best with nerve blocks.[141-149] For example, Najarian et. al. report retrospective results in 289 patients undergoing breast cancer surgery in which some received regional anesthesia (paraverterbral block) while others had general anesthesia. Patients given regional anesthesia had less early post-operative pain, along with less nausea and vomiting.[28] Furthermore, a meta-analysis of 1,404 abstracts and 100 full studies showed that regional analgesia provides better postoperative analgesia than systemic analgesia, regardless of analgesic agent, location of catheter placement, and type and time of pain assessment.[150]

Consistent with the importance of good acute analgesia, a number of studies in thoracotomy patients have shown that regional anesthesia decreases the incidence and intensity of chronic pain.[151,152] For example, a study by Kairaluoma, et.al. reported that regional anesthesia [preincisional paravertebral block (PVB)] provides better immediate postoperative analgesia after breast cancer surgery than opioids.[32] In the same 60 patients, the prevalence and severity of pain at 6-months and 1-year was reduced in those given regional anesthesia.[153] Iohom, et.al. similarly report that among 29 patients who had breast surgery, 80% of the patients given systemic analgesia experienced chronic post surgical pain (CPSP) whereas none did who received regional analgesia.[133] A retrospective analysis among 220 patients who had Caesarean deliveries showed that patients with persistent pain were more likely to have had general rather than spinal anesthesia.[154] Regional anesthesia appears to reduce chronic post-surgical pain[135,137,155-162] in a variety of surgical procedures including herniorrhaphy[163] and vasectomy. Other studies, though, fail to identify any benefit.[164,165] Available data are mostly retrospective; a large prospective trial has yet to be published.

Study Objectives

Cancer surgery promotes metastases by the following mechanisms: 1) immune suppression from surgery *per se*; 2) immune suppression by general anesthetics; 3) opioid-induced immune suppression; and 4)

direct promotion of tumor proliferation and angiogenesis by morphine and stress hormones. Because regional anesthesia and analgesia block the neuroendocrine response to surgery and obviate the need for postoperative opioids, regional techniques may reduce metastatic risk during cancer surgery. This overall mechanism is shown in Fig. 6. Our preliminary results (Section C, below) are consistent with this theory: paravertebral anesthesia and analgesia reduced the recurrence rate after breast cancer surgery by 79%. We note that this reduction was observed even though all patients were given general anesthesia.

We propose to determine whether recurrence rates after breast cancer surgery are reduced in patients who have intraoperative and postoperative regional anesthesia and analgesia, combined with propofol sedation or light anesthesia compared with those who receive intraoperative volatile general anesthesia and postoperative opioid analgesia. We will simultaneously evaluate the effects of regional analgesia on chronic post-surgical pain and quality-of-life.

Fig. 6. Cancer surgery is usually performed under general anesthesia with postoperative analgesia provided by opioids. The stress of surgery, general anesthetics, and opioid analgesics are all immunosuppressive and specifically inhibit cell-mediated immunity that is critical for control of minimal residual disease. Opioids and stress also increase angiogenesis, which promotes growth of cancer cells. Regional anesthesia and analgesia blocks the neuroendocrine stress response, decreases or eliminates the need for general anesthetics, and obviates the need for postoperative opioids.

Regional techniques such as paravertebral and epidural anesthesia/analgesia are easy to implement, inexpensive, and no riskier than general anesthesia. If we were able to demonstrate that regional techniques improve the prognosis of patients undergoing breast cancer surgery, increasing the use of regional blocks would be a simple change in anesthetic practice.

C. Preliminary Results

Breast Cancer

We examined the medical records of 129 consecutive patients undergoing mastectomy and axillary clearance for breast cancer between September 2001 and December 2002.

50 patients had surgery with paravertebral anesthesia and analgesia combined with general anesthesia and 79 patients had general anesthesia combined with postoperative morphine analgesia. The follow-up time was 32±5 months (mean±SD). There were no significant differences in patients or surgical details, tumor presentation, or prognostic factors. Recurrence and metastasis-free survival, with multivariate analysis, was 94% (95% CI 87,100) versus 82% (74, 91) at 24 months, and 94 (87, 100) versus 77 (68, 87) at 36 months in the paravertebral and general anesthesia patients, respectively, P=0.013 (Fig. 7).

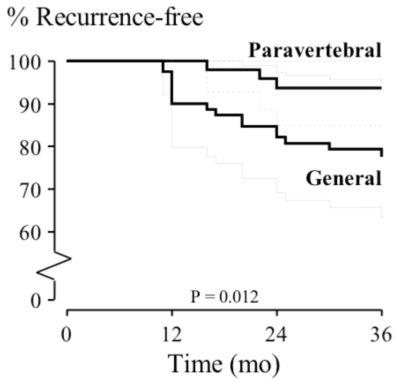

Fig 7. Association between paravertebral block and cancer recurrence (P=0.013); P= 0.012 (log-rank test) in a multivariable model that adjusts for histological grade and number of axillary nodes.

This retrospective analysis suggests that paravertebral anesthesia and analgesia for breast cancer surgery markedly reduces the risk of recurrence or metastasis during the initial years of follow-up. The full paper describing this study was published in *Anesthesiology*.[166]

Prostate Cancer

We evaluated the recurrence of prostate cancer after open radical prostatectomy in patients who received either general anesthesia combined with epidural analgesia or general anesthesia and postoperative opioid analgesia. Patients had either general anesthesia with postoperative opioid analgesia (n=123) or general anesthesia combined with epidural analgesia (n=102). Our major outcome was incidence of metastatic spread or local prostate cancer recurrence through October 2006.

After adjusting for tumor size, Gleason score, preoperative prostate specific antigen status (PSA), and margin, the epidural plus general anesthesia group had an estimated 61% (95% CI 37% to 79%) lower risk of recurrence compared with the general anesthesia plus opioids group, with a corresponding hazard ratio of 0.39 (95% CI 0.21 to 0.73; *P*=0.0003) in a multivariable Cox regression model (fig. 8). Gleason score and baseline PSA were also strong independent predictors of recurrence (hazards ratios of 1.32 [1.12, 1.56], *P*=0.001, and 1.02 [1.00, 1.04], *P*=0.020, respectively).

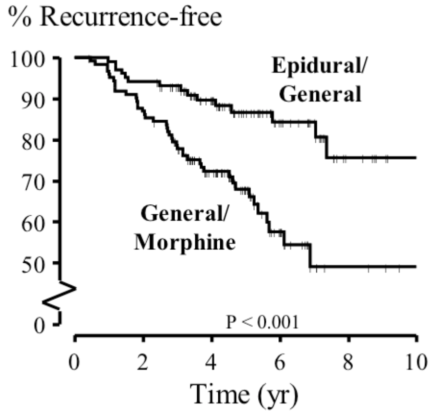

Fig 8. Kaplan-Meier recurrence-free survival estimates for 123 patients given general anesthesia and postoperative opioids (General/Opioid) and for 102 patients given general anesthesia combined with epidural analgesia (Epidural/General) during radical prostatectomy for prostate cancer; univariable *P* value < 0.001. Vertical tick marks represent censored values.

In patients having open prostatectomy surgery with general anesthesia, substituting epidural analgesia for postoperative opioids substantially reduced the risk of cancer recurrence. The full paper describing this study was published in *Anesthesiology*.[167]

Transfusion of Older Blood Promotes Cancer Recurrence

While surgery remains the primary treatment for most cancers, minimal residual malignancy is common. Whether residual tumor progresses to clinical metastases is largely a function of host defense. Blood transfusions are immunosuppressive and increase recurrence risk after cancer surgery. Prolonged storage degrades transfused cells, perhaps augmenting immunosupression. We therefore tested the hypothesis that prolonged red cell storage duration increases the risk of cancer recurrence after colorectal cancer surgery.

We examined data from patients given red cell transfusions for colorectal cancer surgery between 1992 and 2005; 218 patients were given 557 units of blood stored for 12 or fewer days ("Newer tercile"), 194 patients were given 441 units of blood stored between 12 and 17 days ("Middle tercile"), and 229 patients were given 632 units of blood stored 17 days or longer ("Older tercile"). Disease-free survival was estimated with Kaplan-Meier methods. Median [1st quartile, 3rd quartile] storage duration for the three groups were 9 [8, 11]; 14 [13, 15]; and 22 [19, 28]; respectively. Maximum storage duration was 42 days. Each of the disease-free survival curves differed significantly (Fig. 8).

Fig 8. Kaplan-Meier recurrence-free survival estimates for 218 patients given 557 units of blood stored for 12 or fewer days ("Newer tercile"), 194 patients given 441 units of blood stored between 12 and 17 days ("Middle tercile"), and 229 patients given 632 units of blood stored 17 days or longer ("Older tercile"). Each of the disease-free survival curves differed significantly.

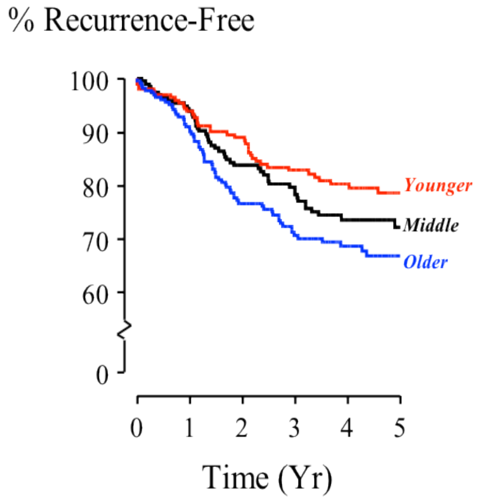

The risk for colorectal cancer recurrence increased significantly and progressively with older blood. Restricting the storage duration of transfused blood may reduce the risk of recurrence after colorectal cancer. These unpublished data provide additional evidence that perioperative management choices have long-term effects on the risk of cancer recurrence.

Tumorigenic and Anti-tumorogenic Cytokine Concentrations

We tested the hypothesis that patients who receive combined propofol/paravertebral anesthesia with paravertebral analgesia exhibited reduced serum levels of protumorigenic cytokines and elevated levels of antitumorigenic cytokines compared to patients who receive sevoflurane general anesthesia with opioid analgesia. We simultaneously tested the hypothesis that matrix metalloproteinase (MMP) concentrations (pro-tumorigenic factors) are increased more with sevoflurane than paravertebral anesthesia.

Primary breast cancer surgery patients were randomly assigned to Propofol/Paravertebral (n=15) or Sevoflurane/Opioid (n=17) anesthesia. Pre- and post-operative serum cytokine and matrix metalloproteinase concentrations were measured. The protective cytokine IL-10 increased more with paravertebral analgesia; IL-8, IL-1ß, MMP-9, and MMP-3 all increased significantly more with sevoflurane/opioid anesthesia (Table 1).

Table 1. Serum Cytokine and Metalloproeinase Concentrations as a function of Anesthetic Technique.

	Sevoflurane/Opioid % Change (n=17)	Propofol/Paravertebral % Change (n=15)	P Value
**IL-10**	-5.5 ± 8.8	190 ± 82	0.02
**IL-8**	50 ± 8.9	-6.0 ± 10	0.02
**IL-1ß**	-10.0 ± 2.2	-30 ± 6.5	0.004
**MMP-9**	87 ± 16.6	28 ± 10	0.006
**MMP-3**	82 ± 37	-6.0 ± 10	0.03
**MMP-1**	-11 ± 5.6	-1.0 ± 9.9	0.39

Values are reported as the means ± SDs.

These data indicate that propofol/paravertebral anesthesia and analgesia help preserve the immune defenses, and potential resistance to tumor progression, metastasis, and recurrence.

Propofol Reduces and Morphine Increases Breast Cancer Cell Migration and Proliferation in vitro

We tested the hypothesis that propofol reduces and morphine increases *in vitro* proliferation and migration of two breast cancer cell lines, each with different metastatic potential.

MDA-MB-231 (estrogen receptor negative) and MCF7 (estrogen and progesterone receptor positive) breast cancer cells were incubated with anaesthetic drugs using clinically relevant concentrations of propofol (1-10 µg/ml) and morphine (10-100 µg/ml). Cell proliferation was determined with CellTiter 96 Aqueous One Solution Cell Proliferation Assay. Cell migration was determined using a Chemotaxis 96-well Migration Assay.

Propofol decreased cell proliferation in both cell lines by up to 22% in MDA-MB-231 cell line and 44% in MCF7. Morphine increased overall cell proliferation in both cell lines by up to 18% in MDA-MB-231. Propofol reduced cell migration by up to 31% in MDA-MB-231 and 73% in MCF7. Morphine increased migration by up to 105% in MDA-MB-231 and by 25% in MCF7. Each effect was statistically significant.

Enhanced proliferation and migration of breast cancer cells with morphine, and reduced proliferation and migration with propofol, suggests that combining a regional block with propofol for cancer surgery may reduce metastatic risk compared with general anesthesia and opioid analgesia.

Paravertebral Blocks Prevent Sevoflurane-induced Breast Cancer Proliferation

We tested the hypothesis that serum from patients given paravertebral analgesia and propofol for breast cancer surgery reduces migration and proliferation of breast cancer cells *in vitro* compared with serum from patients given sevoflurane anesthesia and morphine.

Primary breast cancer surgery patients (n=22) were randomised to receive either propofol/paravertebral anaesthesia and paravertebral analgesia (Propofol/Paravertebral, n=11) or sevoflurane general anesthesia with opioid analgesia (Sevoflurane/Opioid, n=11). The estrogen-receptor negative MDA-MB-231 cell line was treated with patient serum from each group, and the effects on cell proliferation (measured by MTS assay) and migration (wound closure and chemotaxis migration assay) were measured.

The treatment groups were well-balanced for age, weight, surgical procedure, and cancer pathology. Pain scores were lower at 1 and 2 hrs with paravertebral analgesia than with morphine, but were similar at 24 hours. Pain scores and opioid consumption were significantly lower in the Propofol/Paravertebral group. Compared with preoperative values, proliferation of MDA-MB-231 cells treated with postoperative patient serum at 10% concentration from the Propofol/Paravertebral group was significantly reduced compared to the Sevoflurane/Opioid group (-24% vs. 73%, p=0.01). There was no significant change in MDA-MB-231 migration.

Paravertebral analgesia, combined with propofol, prevents the increase in breast cancer cell proliferation that normally accompanies sevoflurane anesthesia. Regional analgesia may thus help reduce recurrence of breast cancer after potentially curative surgery.

D. Research Design & Methods

We propose to compare recurrence rates in patients with breast cancer who will be randomly assigned to paravertebral or thoracic epidural anesthesia and analgesia or to general anesthesia followed by opioid analgesia. This multi-center protocol will be coordinated by the OUTCOMES RESEARCH Department at the Cleveland Clinic.

Regional anesthesia and analgesia help maintain perioperative immune function by reducing general anesthesia requirements, by ameliorating the stress response to surgery, and by sparing postoperative opioids. We propose a prospective randomized trial to determine the effects of paravertebral or thoracic epidural anesthesia and analgesia on breast cancer recurrence. Specifically, we will test the following hypotheses:

> **Primary Hypothesis:** *Recurrence of local and metastatic cancer after primary breast cancer surgery is lower in patients randomized to paravertebral or thoracic epidural anesthesia and analgesia with propofol sedation than to sevoflurane general anesthesia and opioid analgesia.*
>
> **Secondary Hypotheses:** *Chronic post-surgical pain is reduced by paravertebral or thoracic epidural analgesia.*

The background[168] and methodology[169] for our proposed trial have been published. The study is registered at ClinTrials.gov: NCT00418457.

Setting and Population

Patients meeting the following enrollment criteria will be considered:

1) Primary breast cancer without known extension beyond the breast and axillary nodes (i.e. believed to be Tumor Stage 1-3, Nodes 0-2) as determined according to the NCI stage definitions: (http://www.cancer.gov/cancertopics/pdq/treatment/breast/HealthProfessional/page3/print);
2) Scheduled for unilateral or bilateral mastectomy with or without implant
3) Wide local excision (lumpectomy) with node dissection;
4) Written informed consent, including willingness to be randomized to general anesthesia and opioid analgesia or regional analgesia/analgesia.

Exclusion criteria will include:

1) Previous surgery for breast cancer (except diagnostic biopsies and guide-wire insertion);
2) Inflammatory breast cancer;
3) Age <18 or >85 years old;
4) Scheduled free flap reconstruction;
5) ASA Physical Status ≥4;
6) Any contraindication to paravertebral or epidural anesthesia and analgesia (including coagulopathy, abnormal anatomy);
7) Any contraindication to midazolam, propofol, sevoflurane, fentanyl, or morphine
8) Other cancer not believed by the attending surgeon to be in long-term remission;
9) Systemic disease believed by the attending surgeon to present ≥25% two-year mortality.

Review of recent records indicates that ≈1,400 patients per year will qualify for the study based on our inclusion and exclusion criteria (Table 3). We very conservatively expect to recruit 150 patients the first year 200 the second year, and 250 patients per year thereafter for a 5-year accrual of 1,100 patients (see Sample Size Considerations below). Our current enrollment (Table 2) suggests that these estimates are realistic. The study will thus meet enrollment targets if but 20% of available patients participate.

Table 3. Annual Number of Qualifying Patients at Participating Sites.

Institution	Site Director	Available Patients/year
Cleveland Clinic (Main campus, Hillcrest, and Fairview)	Andrea Kurz, M.D.	450
Mater Misericordiae University Hospital, Dublin, Ireland	Donal Buggy, M.D.	180
London Health Science Centre, Ontario, Canada	Sugantha Ganapathy, M.D.	325
Hospital Italiano, Buenos Aires	Adrian Alvarez, M.D.	280
University of Vienna, Austria	Edith Fleischmann, M.D.	175
**All sites**	**Total**	**1410**

Protocol

Patients will be premedicated with 0-3 mg IV midazolam and 0-2 µg/kg fentanyl. Prophylactic antibiotics will be given per surgical routine. Patients will be randomly assigned to paravertebral or epidural anesthesia and analgesia with propofol sedation or to sevoflurane general anesthesia after they have met the

inclusion/exclusion criteria and consent to the study. Computer-generated assignments (using Proc Plan in SAS statistical software) will be stratified by study site. Randomization will be web-based, using a system designed and controlled by the Department of Quantitative Health Sciences at the Cleveland Clinic (out of the control of any investigator). Patients will be randomized after consent is obtained, shortly before surgery.

General Anesthesia and Opioid Analgesia

In patients assigned to general anesthesia and opioid analgesia (General Anesthesia Group), general anesthesia will be induced with 1-3 μg/kg fentanyl and 2-4 mg/kg propofol. Tracheal intubation will be facilitated by succinylcholine or a non-depolarizing muscle relaxant; alternatively, a supraglottic airway (such as a laryngeal mask) will be used. Additional non-depolarizing muscle relaxant will be administered as deemed necessary by the attending anesthesiologist.

Anesthesia will be maintained with sevoflurane in 80% oxygen, balance nitrogen, and fentanyl. Sevoflurane and fentanyl administration will be adjusted to maintain blood pressure and heart rate within 20% of pre-operative values. The lungs will be mechanically ventilated to maintain end-tidal PCO_2 near 35 mmHg. Because hypothermia impairs immune function, normothermia[170-173] — a distal esophageal temperature of 36°C — will be maintained with forced-air warming.[174]

Intravenous morphine sulfate will be titrated to a respiratory rate of 12-14 breaths per minute near the end of surgery. When surgery is complete, muscle relaxant will be antagonized, if necessary, and the trachea extubated. Post-operative analgesia will be morphine, provided as needed IV or via patient-controlled pump. The initial pump setting will be for 1-mg boluses with a 6-minute lockout period and no background infusion. Additional morphine will be provided as necessary to maintain good pain control, either as needed or by changing the pump settings. Morphine will be the first-line drug; but hydromorphone will be substituted at one-fifth the morphine dose in patients who do not tolerate morphine. After approximately 24 hours, patients will be transitioned to acetaminophen and non-steroidal anti-inflammatory analgesics; oral opioids will also be permitted if necessary. Nearly all patients tolerate morphine. Bio-availability and individual responses of other opioids differ from patient to patient,[175-178] but can be converted into morphine equivalents.[179] This approach is used routinely in analgesia studies.

Regional Anesthesia and Analgesia

In patients assigned to paravertebral or epidural anesthesia & analgesia (Regional Analgesia Group), analgesia will be provided by paravertebral or epidural blocks.[28-32,180] Paravertebral anesthesia will be provided either with a thoracic (T) interspace 2-4 catheter or multi-level injections from thoracic interspace 1 to 5, or as clinically appropriate depending on the anticipated scope of surgery.[32] When a catheter is used, it will be inserted into the ipsilateral paravertebral space at the level of T2/3 or T3/4 using a standard technique.[181] Briefly, patients will be positioned laterally, curled so their knees approach the chest. The upper thoracic spinous processes (T1) will be identified with a marking pen. Opposite these, a series of points, 3 cm lateral to the midline, will be marked ipsilateral to the site of surgery. A vertical line between these points, parallel to the midline, corresponds to the vertebral transverse processes. Under aseptic conditions, a Tuohy needle will be advanced to contact the transverse process at a depth of 3-4 cm. The needle will then be “walked off” the caudal surface of the transverse process and further advanced 1-2 cm. Loss of resistance to saline indicates passage of the needle through the costo-transverse ligament into the paravertebral space. A multi-lumen paravertebral catheter will then be advanced into the space 3-4 cm. Each patient will be given a 10 to 20-ml bolus of 0.5% bupivacaine or 0.5% ropivacaine with epinephrine after a test dose with 1.5% lidocaine and 1:200,000 epinephrine. Near the end of surgery, an infusion of 6-10 ml/h of either solution will be started; the infusion rate will be reduced or increased as deemed necessary by the attending anesthesiologist. Local anesthetic infusion will continue as clinically necessary, but not longer than 48 hours; the catheter will be removed before hospital discharge.

When a multi-level technique is to be used, separate injections at thoracic interspace 1 to 5 will be performed with a graded 22-gauge Tuohy needle. The needle will be inserted 2.5 cm lateral to the superior aspect of the spinous process on the ipsilateral side of surgery and "walked off" the transverse process in a caudal direction 1 cm distal to the transverse process. Ropivacaine 0.75%, 5 ml, will be given at each of the 5 levels via extension tubing attached to the syringe. Additionally, injections of ropivacaine 0.5% will be given by

the surgeon to block cervical and contralateral thoracic nerves that also contribute to the innervation of the breast. A comparable dose of bupivacaine may be substituted.

Paravertebral anesthesia will be supplemented with propofol (usually infused at a rate of 60-90 μg/kg/min) which can also be administered during insertion of the block. However larger amounts can be given per discretion of the attending anesthesiologist depending on the adequacy of the block, extent and duration of surgery, and patient cooperation. We expect that most patients will be able to breathe spontaneously with nasal cannula oxygen; however, a laryngeal mask can be inserted if necessary in patients receiving larger propofol doses. Sevoflurane and/or opioids can be given at the discretion of the attending anesthesiologist if clinically required; similarly, an endotracheal tube can be inserted if necessary. However, we expect that volatile anesthesia and intubation will rarely be required in patients assigned to paravertebral anesthesia & analgesia.

When epidural anesthesia is chosen, a T4 epidural catheter will be inserted using a standard technique. If T4 insertion proves difficult, catheter insertion will be attempted at T3 or T5. After negative aspiration for blood, patients will be given a test dose of 3 ml of 1.5% lidocaine and 1:200,000 epinephrine. The catheter will be re-inserted or repositioned as necessary until both aspiration and test dose are negative. Each patient will be given an additional 3-ml bolus of the same solution to provide intraoperative analgesia. The catheter will be repositioned or reinserted as necessary if a sensory block to temperature cannot be confirmed in the surgical dermatomes. Additional 3-ml boluses of 0.5% bupivacaine or 0.5% ropivacaine with epinephrine will be given hourly during surgery to maintain anesthesia; additional boluses will be permitted at the discretion of the attending anesthesiologist.

Postoperatively, patient-controlled epidural analgesia will be provided by an infusion of ropivacaine 0.1-0.2% and 2 μg/ml fentanyl with epinephrine or similar solution that will start shortly before the patient emerges from general anesthesia. The infusion basal rate will be 4-8 ml/hour with 2 ml per demand and a lockout period of 20-30 minutes. The local anesthetic concentration, infusion rate, and demand volume will be adjusted as clinically indicated to provide excellent analgesia while avoiding hypotension.

Postoperatively, analgesia will be primarily provided by the regional block. It will be supplemented with acetaminophen and/or NSAIDs if needed, or *per* individual sites' routine protocol. However, supplemental morphine will be provided if pain relief is inadequate, either "as needed" or by patient-controlled infusion. As soon as practical, usually at about 24 hours, patients will be transitioned to acetaminophen and/or non-steroidal analgesics and, if necessary, oral opioids. In our experience, patients given epidural or paravertebral analgesia rarely require intravenous opioids, and few even require oral opioids. Catheters, if used, will be removed before hospital discharge, but no later than the second postoperative day. Similar local anesthetics, or combinations of local anesthetics may be substituted by the attending anesthesiologist.

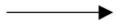

Fig. 9. Trial Profile.

All anesthesiologists participating in the study will have experience with high thoracic or paravertebral blocks and have done at least ten recent non-study cases. But even in experienced hands, we can anticipate that 5-10% of the blocks will fail (the failure rate was 5% in our preliminary study) and our sample-size estimate (below) compensates for patients crossing over from regional to general anesthesia. Patients with unsuccessful blocks will be switched too the alternative regional technique if practical, or given general anesthesia and morphine postoperative analgesia as described above. The overall trial profile is shown in figure 9.

Fluid Management

The primary fluid management will be lactated Ringer's or similar crystalloid solution. Up to 500 ml will be given with induction of anesthesia at the anesthesiologist's discretion. Subsequently, lactated Ringer's solution will be given at a rate of 2-4 ml/hr. Blood loss will be replaced with lactated Ringer's solution at a 3:1 ratio, colloid at a 2:1 ratio, or red cells at a 1:1 ratio. It is unlikely that many patients having breast surgery will require blood transfusions. However, red blood cell transfusions are immunosuppressive, an effect that is only partially ameliorated by leukocyte filtering. Red cell transfusions will thus be strictly controlled by protocol. Target minimum hematocrit (HCT) will thus be determined *prospectively* (before randomization) based on the patient's age and cardiovascular status. Boluses of ephedrine (5 mg) or neosynephrine (100 µg) will be given as necessary at the discretion of the attending anesthesiologist.

The target HCT will be 26% in patients aged <65 yr having no significant cardiovascular disease. The HCT will be maintained at 28% or above in patients aged ≥65 yr *or* having cardiovascular disease. Significant cardiovascular disease will be defined as previous myocardial infarction, angina, congestive heart failure, cardiomyopathy, hypertension requiring treatment (or having a diastolic blood pressure exceeding 90 mmHg), or peripheral vascular disease. HCT will be maintained ≥30% in patients having both cardiovascular disease and an age ≥65 yr. *Leukocyte-depleted* allogeneic blood will be administered only as necessary to maintain the prospectively-determined target HCTs.

Prophylaxis for postoperative nausea and vomiting will be provided per clinical routine, typically 4 mg of ondansetron. Rescue treatment will be provided per clinical routine with additional ondansetron, metacloprimide, droperidol, or other agents. However, dexamethasone will be specifically avoided since the drug is immunosuppressive.

In all cases, good clinical judgment will predominant and the attending anesthesiologist will modify the protocol as necessary to provide optimal and safe care. However, the proposed methods are routine and we expect modifications to be necessary only rarely.

Measurements

Morphometric and demographic characteristics of the patients in each treatment group will be tabulated. For follow-up purposes, we will obtain contact information for the patient, a family member, and the names of the patient's oncologist and primary physician. The date of the last menstruation will be recorded in pre-menopausal women since cycle phase influences natural killer cell function[74] and metastasis risk.[182,183] Perioperative use of beta-blockers and COX inhibitors will be recorded since both impact the immunosuppressive and tumor-promoting effects of surgery.[66]

Anesthetic data in the patients given general anesthesia will include volatile anesthetic dose in MAC-hours. In the patients given regional anesthesia, upper and lower block levels, the type of anesthesia (paravertebral vs. epidural), and the initial dose of local anesthetic will be recorded — as will the amount given via infusion over the first 48 postoperative hours. In both groups, anesthetic depth will be estimated using the Bispectral Index (BIS, Aspect Medical, Newton, MA), which is by far the best-validated monitor of anesthetic effect. BIS will be recorded at no longer than 15-minute intervals. But in most cases, BIS will be electronically down-loaded from the monitors where the signal is automatically recorded at 1-minute intervals. The software version will also be recorded.

BIS is a processed EEG parameter that results from a multivariate discriminate analysis of the time domain, frequency domain, and high order spectral sub-parameter features of the EEG.[184] The electroencephalographic (EEG) Bispectral Index has proven to be a quantifiable measure of the effects of anesthetic agents on the central nervous system and it can be related to the hypnotic component of the anesthetic state. BIS is a dimensionless number scaled from 0 to 100, with 0 being complete brain electrical silence and 100 representing an awake EEG.[185] A BIS index of 40-60 is regarded as adequate surgical anesthesia. BIS is a reliable, predictable, convenient and practical means of measuring the level of consciousness[186] and is therefore a suitable surrogate to the depth of anesthesia. More than 800 publications related to BIS are listed at the National Library of Medicine and BIS is the only system that has been shown to reduce recall of intraoperative events.[187]

Upper and lower sensory block levels (in terms of dermatomes and pin-prick) will be recorded in the regional patients before surgery and shortly after arriving in the post-anesthesia care unit.

In both groups, the total dose of propofol and other drugs (including antiemetics) will be recorded. Blood loss will be recorded, along with fluid administration including allogeneic blood. Blood pressure and heart rate, will be recorded at 15-minute intervals final intraoperative core temperature will also be recorded.. Total fentanyl and morphine use during surgery will be recorded, as will morphine use over the first 48 postoperative hours; morphine use will be broken into the first two postoperative hours, from two hours until the first postoperative morning, and from the first to second postoperative morning. For analysis purposes, other postoperative opioids will be converted into equivalents of morphine sulphate using factors in Principles of Analgesic Use in the Treatment of Acute and Chronic Cancer Pain.[179] Patients will be asked to rate their pain on an 11-point Likert scale after one and two hours of recovery, 24 hours after surgery, and on the second postoperative morning. Complications related to the assigned anesthesia and analgesia will be recorded.

Postoperative nausea and vomiting will be evaluated after two hours of recovery, and on the first and second postoperative mornings. Patients will be asked if they had any nausea. Patients will also be asked about any emetic episodes, defined by vomiting or retching. As the mechanism of retching and vomiting is similar, and the latter is much more frequent, retching is often considered as vomiting (without expulsion of gastric contents). For the sake of simplicity we will therefore consider retching and vomiting synonymous. The duration of hospitalization will be recorded.

If patients are no longer in the hospital by the second postoperative morning, they will be called by an investigator who will query them about pain and opioid use. Clinical experience suggests that nearly all patients will tolerate morphine. But if clinically necessary, other opioids will be used at equivalent doses as shown in Table 4. Bio-availability and individual responses differ from patient to patient.[175-178] Nonetheless, the following table, adapted from Principles Of Analgesic Use In The Treatment Of Acute And Cancer Pain (Fifth

Edition) 2003,[179] is a reasonable basis for converting various opioids into morphine equivalents in the perioperative period. Piritramide[188,189] is included because the drug is commonly used in Europe.

Table 4. Opioid Equivalents.

	Oral Dose (mg)	Parenteral Dose (mg)
**Morphine**	30	10
**Hydromorphone**	7.5	2
**Oxycodone**	20	-
**Methadone**	10	5
**Levorphanol**	4	2
**Oxymorphone**	-	1
**Meperidine**	300	75
**Nalbuphine**	-	10
**Butorphanol**	-	2
**Pentazocine**	50	30
**Buprenorphine**	-	0.4
**Piritramid**	-	15

Prognostic factors related to the risk of breast cancer recurrence will include details of the tumor size, grade, type, and estrogen receptor status; the extent of axillary nodal disease; and whether preoperative or postoperative adjuvant chemotherapy and radiotherapy was used. For staging, we will use the 10/21/05 National Cancer Institute TMN Definitions.

When available, we will record the tumor's OncoType DX score (0-50) which predicts the likelihood of distant recurrence. It has been well validated in women with newly diagnosed, stage I or II, node-negative, estrogen receptor-positive breast cancer who will be treated with tamoxifen. The test analyzes the expression of a panel of 21 genes.[190-192]

Additionally, we will compute the Nottingham Prognostic Index, a score for which the prognosis for breast cancer is based on the formula: 0.2 (tumor size) + histological grade (1 = Grade 1, least aggressive histology, Grade 2 = intermediate histology, Grade 3 = most aggressive histology) + axillary lymph node involvement (1 = no nodes involved, 2 = up to 3 nodes involved, 3 = more than 3 nodes involved).[193] A score below 3.4 suggests a good outcome whereas a score between 3.4 and 5.4 suggests an intermediate prognosis. Additionally, we will determine whether the resection margins are clear of tumor.

Cancer recurrence will be evaluated by tracking all patients at six-month intervals throughout the study or until recurrence is documented. Patients and/or their health-care providers will be contacted at each six month follow-up interval for up to ten years to confirm recurrence status, and to obtain details of recurrence if there is one. Yearly mammograms are standard in these patients and abnormal results usually provoke a biopsy. Biopsy results will be obtained whenever possible. The site of initial detected recurrence will be determined.

At each contact, we will determine if additional surgery was required, what the reason was, and what kind of anesthesia was used (general vs. regional vs. monitored anesthesia care). Trained investigators who will be guided by scripts prepared by our social worker will contact patients and their families. All follow-up contact with patients, families, and care-givers will be conducted by investigators who are strictly blinded to group assignment and intraoperative management; questions that might unblind the follow-up investigators will be specifically avoided.

All cases of apparent recurrence will be evaluated by a committee that will make the final determination using all available laboratory and clinical evidence. Members of the Adjudication Committee will be strictly blinded to randomization and actual perioperative management. The Committee will consist of the site directors; however, directors will be excused when cases from their own sites are discussed to keep the process completely blinded. The diagnosis of a first breast cancer recurrence should normally only be made only when both the clinical and laboratory findings meet criteria specified by the National Surgical Adjuvant Breast and Bowel Project Protocol B-39. Suspicious findings do not constitute criteria for breast cancer recurrence.

Quality-of-Life and Chronic Pain Measures

We will evaluate the SF-12 Health Survey (SF-12), the modified brief pain inventory (mBPI), and the Neuropathic Pain Questionnaire Short Form (NPQ-SM) at six-month intervals until the end of the study or recurrence is documented, starting six months after surgery. Official versions of each are available in German.

The **SF-12** is an abbreviated version of the SF-36 Health Survey, a well-established instrument to assess psychological and physical aspects of health related quality of life (QoL).[194] The principal scores from the SF-12 are a physical health composite (PCS-12) and a mental health score (MCS-12). In addition, an eight-domain profile can be produced, providing scores for Physical Function, Role Physical, Bodily Pain, General Health, Vitality, Social Functioning, Role-Emotional, and Mental Health. Theta reliability estimates range from 0.73 to 0.87 across the eight scales, whereas the value for the PCS-12 is 0.89 and that of the MCS-12, 0.86. The SF-12 is thus our choice survey of global health because it is well validated, accurate, precise, and requires less than two minutes to administer.[195]

The **BPI** is a practical method of evaluating pain severity and its impact on patient function. The BPI was created because the McGill Pain Questionnaire is too long and difficult, and doesn't evaluate pain history or the extent to which pain interference with daily activities. The BPI includes four rating pain intensity, and seven covering the impact of pain. Intensity is recorded on an ordinal scale from zero (no pain) to ten (worst imaginable pain). In the impact of pain section, these ratings are made on zero-to-ten numeric scales running from no interference to complete interference.[195-197] The reliability alpha values for the four pain intensity items of 0.87 (N=1,106). Alphas for the interference scale are 0.91. The internal structure of BPI is logically consistent: as ratings of pain intensity increase, the interference items are endorsed in a sequence running from work, to mood, sleep, activity, walking, and finally, relations with others.[195]

The **NPQ-SF** is an abbreviated version of the Neuropathic Pain Questionnaire (NPQ), a clinical tool with the ability to differentiate neuropathic from other types of pain. Stepwise discriminate analysis identified three items: Tingling Pain, Numbness, and Increased Pain due to Touch. The resulting canonical discriminate function was able to predict neuropathic pain wRevRith a sensitivity of 64.5%, sensitivity of 78.6%, and total predictive accuracy of 73%. This straight-forward questionnaire will allow us to use the power of clinical symptoms and signs for purposes effectively identifying patients with neuropathic pain.[198,199]

The English version has a reliability alpha values for the four pain intensity items of 0.87 (N=1,106). Alphas for the interference scale are 0.91 for the English version. Factors analyses have consistently supported the division into severity and interference factors in studies in United States, France, China, and Philippines. The BPI detects expected differences in severity of pain between groups of patients who different in the site of their disease, in their requirements for analgesics, and in the presence of metastases. The correlations among the pain intensity ratings (e.g. now, least, worst, average) fell in the range of 0.57 to 0.80, whereas correlations among the inferences scales ranged from 0.44 to 0.83. Correlations between the intensity and interference ratings ran from 0.27 to 0.63. The internal structure of BPI appears logically consistent: as ratings of pain intensity increase, the interference items are endorsed in a sequence running from work, to mood, sleep, activity, walking, and finally, relations with others.[195]

Because of space constraints, this section only summarizes the data we will collect. More detail is provided in Appendix 4, which contains our case-report forms.

Data Analysis

Our primary outcome is time to metastatic spread or local cancer recurrence, and the secondary outcome is chronic pain. The randomized groups will be descriptively compared on all baseline variables using summary statistics such as mean and standard deviation, median and quartiles or frequency and percent, as appropriate. All tests will be 2-tailed and the significance level for primary analyses will be 0.05. SAS statistical software, Carey, NC, will be used for all data analysis.

Primary analysis

The primary analysis will be intent-to-treat (ITT), where all subjects are analyzed in the group to which they were randomized. We expect that 5% of the regional blocks will fail, and that these patients will then be converted to general anesthesia. Such cases will be analyzed in the group to which they were randomized.

We will first assess the effect of regional versus general anesthesia on time to recurrence of local or metastatic cancer univariably by comparing the randomized groups with Kaplan-Meier analysis and a log-rank test.. Equal precision 95% confidence bands[200] will be constructed and plotted for each of the randomized groups along with the Kaplan-Meier product-limit recurrence-free estimates. As usual for survival analysis, patients lost to follow-up due to uncontrollable factors during the study will be censored at the time of last contact.

Our primary analysis will be a Cox proportional hazards model in which we will assess the effect of regional versus general anesthesia via a hazard ratio (95% CI) for cancer recurrence/metastatic spread while adjusting for the following a-priori determined baseline and perioperative factors: clinical center,TNM, stage, center, age, race, ethnicity, type of surgery, estrogen-receptor status, and whether preoperative or postoperative adjuvant chemotherapy, radiotherapy, or endocrine therapy was used. Postoperative therapies a patient receives after the index surgery (and before a patient's recurrence) will be included via time-varying covariates. These covariables (other than clinical center) are either known or highly suspected to be related to breast cancer recurrence. We will adjust for them regardless of statistical significance in the model order to estimate the treatment effect conditional on these factors. The interaction between treatment effect and clinical center will also be assessed. Postoperative therapies are included since these are such important predictors of outcome, and unrelated to the intervention. All of our interim analyses will use this same covariable-adjusted model.

We choose a covariable-adjusted model as our primary outcome in order obtain a more generalizable treatment effect estimate than would be given with a non-adjusted model. The covariable-adjusted model allows risk calculation for individual patients based on the included characteristics, and averages the treatment effect for covariables not included. Our results will thus be more useful to individual patients and more generalizable to patient populations with perhaps different distributions of the included covariables.[201,202] Our choice is not based on concern for confounding factors (i.e., those related to both the intervention and the outcome), since the randomized groups will likely be well balanced on baseline factors. Although multivariable analysis for a continuous outcome (linear regression) increases the precision of the estimated treatment effect and thus add power to a randomized trial even if perfect balance is achieved, [203] such is not typically the case for survival or binary outcomes.[201,202,204] The Cox regression treatment effect estimate adjusted for strong prognostic variables will tend to be slightly farther from the null effect and have slightly larger or the same standard error as the unadjusted model, usually with slightly improved power.

Interim analyses for efficacy and futility during this group sequential design will be conducted at every 25% of the maximum number of required recurrences. See details in Sample-size Calculations, *Interim Analyses and Stopping Boundaries.*

Secondary analyses

Our main secondary outcome will be chronic pain. We will assess the effect of regional versus general anesthesia separately on pain measures (mBPI and NPQ-SM) and quality of life (Sf-12) using linear mixed effects models, considering patient as random effect, and incorporating the repeated measurements within patient at six month intervals, beginning 6 months after surgery. In these models we will first assess the interaction between treatment and time. Treatment effect will be estimated marginally (collapsing over time) if no interaction observed, and separately at various times in presence of an interaction. Within-patient covariance structure for the mixed effects models will be chosen based on comparing reasonable models on the AIC criterion.

In secondary analyses we will also specifically evaluate the relationship between the bispectral index and cancer recurrence using Cox proportional hazards survival analysis. In particular, we will consider cumulative time with BIS<45, since that has previously been associated with mortality in general surgical

populations, with most of the observed mortality being due to cancer.[205,206] Also, a more sophisticated approach of accounting for the post-randomization variables by considered them as outcomes themselves will be considered, potentially by adapting the methods of Rochon[207,208] to survival analysis. We will also test for interactions between the treatment effect and the following selected covariables: red cell transfusion; tumor size, grade, and type; estrogen receptor status; and whether preoperative or postoperative adjuvant chemotherapy, radiotherapy, or endocrine therapy was used. These analyses may help identify specific subgroups of patients for whom the intervention appears especially helpful or not helpful. Although pre-specified, we will interpret the estimated interactions and their statistical significance with much caution since there are several of them (increased false positive conclusions) and they are not the primary analysis or focus. Our assessment of interactions will be viewed as exploratory and hypothesis generating.

An as-treated analysis will be conducted using the same methods as described above for the intent-to-treat analysis. Because it is well known that as-treated analyses often lead to quite biased estimates of the true treatment effect, these results will be interpreted with much caution and always secondary to the intent-to-treat results.

Analyses will be conducted using SAS statistical software, Cary, NC. The significance level (i.e, Type I error) will be 0.05 for each hypothesis. Accordingly, correction to the significance criterion for multiple comparisons within a hypothesis (eg, comparisons at various time points) will be made in order to maintain the overall significance level.

Sample-size Calculations and Sequential Monitoring

Primary Outcome: Cancer Recurrence

We hypothesize that our control group (general anesthesia with opioid use) will have a pattern of recurrence similar to that observed in the summary of trials reported by Saphner,[209] and that stage will be similar to that for patients previously treated at the Cleveland Clinic. The stage distribution of patients in the Cleveland Clinic Breast Center Registry for women diagnosed and/or initially treated at the Cleveland Clinic between 1998 and 2002 was N=1097 (49.7%) Stage I, N=956 (43.3%) Stage II, and N=156 (7%) Stage III. For the combined 3,585 patients across seven Eastern Cooperative Oncology Group studies, Saphner et.al. reported the hazard of recurrence (percent recurring) for each year of follow-up as: year 0-1= 7.7, year 1-2=13.3, year 2-3=11.9, year 3-4=9.0, year 4-5=6.7, year 5-6=4.5 and year 6-7=4.7. These hazard estimates may be somewhat conservative because the Saphner data include stage 0 and stage IV patients (a lesser percentage) and our study will not include such patients. Our narrower population at both spectrum ends is expected to give similar estimates.

Given the above hazard rates for the general anesthesia group, we would need to observe a maximum of 356 recurrences to have 85% power at the 0.05 significance level to detect a 30% reduction in the risk of cancer recurrence (i.e., hazard ratio 0.70), allowing for 3 interim analyses at 25%, 50% and 75% of the maximum number of events, plus a final analysis. These calculations include a 3% dropout rate per year and the assumption that 5% of the regional blocks will fail (and thus be converted to general anesthesia which dilutes the treatment effect by 5%, although analyzed as intent-to-treat). We further assume that we will enroll 150 patients the first year, 200 the second year, and 250 patients per year for an additional three years; the total will thus be 1,100 patients over five years of enrollment.

With the expected accrual and assumed hazard rate for the general anesthesia group, and under the alternative hypothesis of 30% treatment effect, the interim analyses would occur at 3.6 years (89 events), 5.0 years (178 events) and 6.3 years (268 events), with a final analysis, if needed, at 8.3 years (356 events, Fig. 10). As usual for survival statistics, the interim analyses are based on the number of recurrence events, not enrollment or elapsed time. Under the alternative hypothesis, the probabilities of stopping the trial for either efficacy or futility at the first, second or third interim analyses are 0.07, 0.26, and 0.37, with a probability of 0.30 of continuing to the final look. Larger true treatment effects would have larger probabilities of stopping for efficacy before the final look.

Our calculations assume non-binding stopping rules (the Data and Safety Monitoring Board will have ultimate authority) and account for monitoring both the null and alternative hypotheses. We use the gamma family spending function of Hwang, Shi, and Decani (1990),[210] where the parameter gamma controls how fast

alpha or beta is spent throughout the trial. We use gamma=-4 for efficacy (i.e., alpha spending), which closely resembles the O'Brien-Fleming spending function, and gamma=-2 for futility (i.e., beta spending), which is between the Pocock and O'Brien-Fleming approaches (Fig 10). We are thus spending the beta somewhat faster than the alpha during the trial. The boundaries meet at the end of the maximum accrual, so that a decision for either efficacy or futility will be made at some point during the trial (Fig. 11). Stopping boundaries for efficacy (and futility in parentheses) are as follows (Fig 12): first look P ≤ 0.0016 (P > 0.9478); second look P ≤ 0.0048 (P > 0.7136); third look P ≤ 0.0147 (P > 0.2429); last look P ≤ 0.044 (P > 0.044). These power analyses are conservative since they are based on a univariable analysis, whereas our primary covariable-adjusted analysis will presumably have somewhat higher power.

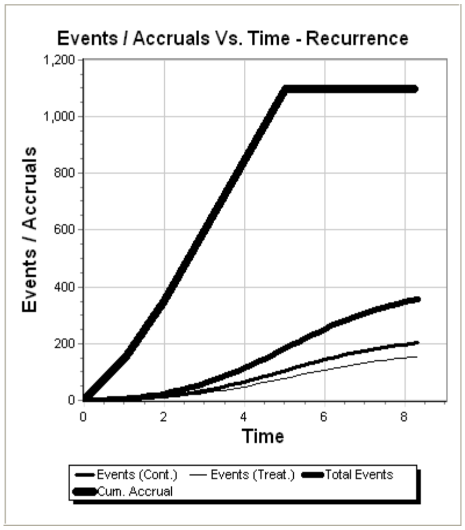

Figure 10

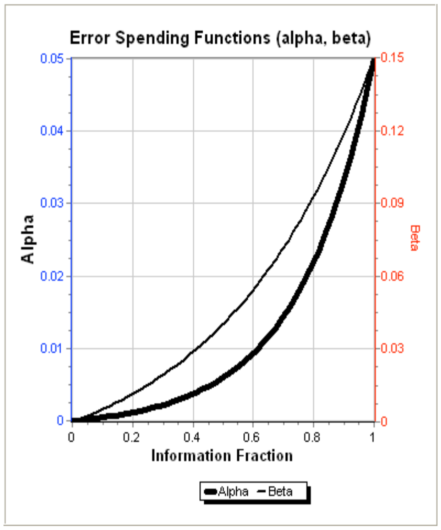

Figure 11

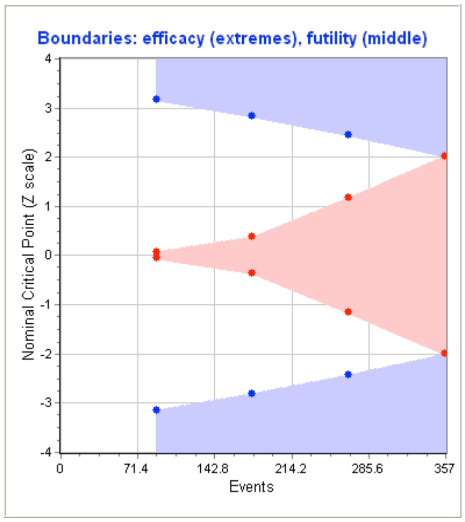

Figure 12

Data Management and Quality Assurance

The proposed study will be registered with ClinicalTrials.Gov before the first patients are enrolled. We will also publish a "methods" paper that will identify important aspects of the protocol and our *a priori* outcomes.

The trial will be directed by an Executive Committee consisting of Daniel I. Sessler, M.D., Donal Buggy, M.D., and Andrea Kurz, M.D. Our principal statistician, Edward Mascha, Ph.D., will attend Executive Committee meetings in a non-voting capacity. Principal data quality and auditing personnel will be available should the Committee need to meet with them.

The Executive Committee will evaluate all results from the proposed trial as the Committee deems it necessary. It will be the responsibility of this committee to alert the IRB *via* letter to any untoward toxicity in one of the study groups. This committee, along with the IRB, will have exclusive authority to stop the study either because the hypotheses have been confirmed or denied, or because adverse events are detected.

All interviewers will undergo training and must demonstrate a high level of proficiency before being certified to interview subjects. Procedures used to assure the integrity of data include (1) quarterly external audits by **O**UTCOMES **R**ESEARCH staff, (2) data entry procedures following standard operating procedures (SOPs), and (3) data queries and resolution processes following SOPs. Frequent interaction among members of the study Executive Committee (PI, co-investigators, consultants), RAs, and others as necessary, will maintain overall quality assurance. They will meet or have conference calls at least quarterly throughout the data collection period, and as necessary thereafter. High-resolution, full-duplex streaming video conferencing is already available at most study sites including Cleveland, Louisville, Bern, and Vienna.

Hard-copy forms will be stored in locked cabinets within a secured area. To protect electronic records and files against loss, duplicate files will be maintained and on Division of Anesthesiology servers at the Cleveland Clinic. These servers are highly secured because they already contain much patient-related

information and are backed up daily to tape that is maintained in a remote location. The system fully meets all applicable HIPAA Privacy and Security rules. Access to the database and backups are strictly monitored according to need.

At least 80% of the data will be independently audited to confirm consistency among patient records, study data sheets, and the main database. Data will be maintained on a custom-designed FileMaker or SQL relational database. Data will be transcribed by separate sets of investigators. We have programmed and used similar password-protected databases in our previous major outcome trials.[211-213] Trial management will be coordinated from Cleveland. The PI will visit each site at reasonable intervals. Please see the Manual of Operating Procedures (MOP), Appendix 3, for much additional detail about trial conduct, regulatory management, and operation of the DSMB.

Strength, Limitations, and Significance

Strengths

The mission of the **O**UTCOMES **R**ESEARCH Consortium is to evaluate simple, inexpensive, low-risk interventions that have the potential to markedly improve perioperative outcomes. For example, we have shown that maintaining intraoperative normothermia (at a cost of $8/patient) reduces blood loss[214] and transfusion requirement,[215] decreases the duration of recovery[216] and hospitalization,[170] reduces wound infection risk by a factor of three,[170] prevents most shivering,[217] and improves thermal comfort.[218] Similarly, we have shown that providing supplemental oxygen (at a cost of $0.03/patient) halves the risk of surgical wound infection.[211,219] More recently, we have shown that restricting storage duration of transfused red cells to less than 14 days reduces the relative risk for one-year mortality after cardiac surgery by 30%.[220] The proposed study continues this tradition by asking if a straightforward change in anesthetic management can reduce the risk of cancer recurrence.

The major risk of cancer surgery is neither the surgery *per se* nor the anesthesia: it is recurrence of cancer — which is usually a lethal event. We expect to demonstrate that switching to regional anesthesia and analgesia reduces the risk of cancer recurrence by about the same amount as postoperative radiation or chemotherapy. But this switch is considerably safer and less expensive than radiation or chemotherapy. Confirming our primary hypotheses, that a straightforward change in anesthetic management reduces the risk of cancer recurrence, would be a major advance and would markedly improve the outcome from surgery for breast cancer. Although we propose to evaluate only one type of cancer, it seems likely that the putative benefits of regional anesthesia would apply to other cancer surgery as well.

A strength of our study is that we will evaluate "hard" outcomes (cancer recurrence) rather than intermediate or indicator outcomes. Paravertebral and thoracic epidural blocks are routine techniques and most anesthesiologists are familiar with the methods. In any case, all anesthesiologists could easily learn these blocks if a convincing benefit were demonstrated. Our results will thus be immediately applicable to clinical practice.

We propose to study breast cancer for three reasons. First, this disease is common: breast cancer is the leading type of cancer in women. This high incidence will make recruiting patients easier and speed completion of the study. Just as important, our results will be immediately applicable to large numbers of women without extrapolation — although extrapolation to other forms of cancer would seem reasonable. The second reason to study breast cancer is that it poses an intermediate risk of recurrence and death, an important consideration since regional anesthesia is unlikely to be helpful in tumors that kill nearly everyone (such as lung cancer). Conversely, it would be difficult to demonstrate a benefit for slow-growing tumors that metastasize only in the very latest stage (such as prostate cancer). Our third reason for focusing on breast cancer is that the surgery is amenable to regional anesthesia using techniques familiar to most anesthesiologists.

The proposed study is powered to detect moderately sized treatment effects. We will have an 85% power to detect a treatment effect of only 30%, which is *much* smaller than the one observed in either of our preliminary studies. There is thus little chance of a Type II statistical error; that is, missing a substantive treatment effect. Furthermore, we will be able to accurately characterize the magnitude of any identified

treatment effect. This is an important consideration since clinicians need to know how good proposed treatments are, not just that they are "significant."

The principal investigator has been continuously NIH funded for 19 years. On average, each primary grant resulted in two full papers published in the *NEJM* or *Lancet* (along with >350 others). The team responsible for this work (the OUTCOMES RESEARCH Consortium, www.or.org) will be fully involved in the proposed study and all participating sites are part of the OUTCOMES RESEARCH network. In addition to designing and conducting our own studies, the OUTCOMES RESEARCH Consortium has considerable experience managing large multi-center clinical trials and is currently managing large NIH-funded trials including AG 024040, DE 14543, GM 061655, DE 015760, and DE 016477. And, of course, the Cleveland Clinic has superb infrastructure for clinical research. It is thus highly unlikely that the study team will encounter any insurmountable technical or administrative difficulties with the proposed studies.

Limitations

That a simple change in anesthetic technique might reduce cancer recurrence is an innovative concept, perhaps to the point of seeming unlikely. However, there exists strong *in vitro* and animal data to support our theory. The underlying mechanisms by which general anesthesia and opioid analgesia might promote tumor dissemination are fairly well established. And finally, our preliminary study supports a substantial clinical effect. Given available information and the importance of the question, it is surprising that a randomized clinical trial has yet to be published. Yet to the best of our knowledge, none is in progress or has even been attempted.

It might seem surprising that an effect of anesthesia on cancer recurrence has not been noticed previously. But most centers use either general or regional anesthesia, and outcome differences amongst centers are usually attributed to surgical technique and other center-related effects. Furthermore, cancer recurs years after surgery and would not normally be attributed to anesthetic management. In this regard, an effect of anesthetic management on tumor recurrence may be similar to surgical wound infections which were conventionally attributed to surgical technique, although studies by the OUTCOMES RESEARCH Consortium subsequently demonstrated that two simple anesthetic techniques (maintaining normothermia[170] and providing supplemental oxygen[211,219]) each reduce infection risk by a factor of two or three. Clinicians had noted neither of these benefits and neither intervention was considered to be routine management at the time.

A reasonable question would be whether it might be preferable to first conduct additional retrospective analysis. We queried a number of large databases including those at the Cleveland Clinic, Duke University, and the Center for Medicaid and Medicare Services, but none contains terms for both anesthetic technique and cancer outcome. A retrospective analysis would thus be non-trivial. But more importantly, a chart review will suffer the limitations of any retrospective study including treatment bias, poor control of confounding factors, and inadequate record detail. Thus, the only type of study that can definitively confirm (or deny) our hypotheses is a randomized, blinded trial of the sort we propose.

Both thoracic epidural or paravertebral anesthesia block the neuro-endocrine stress response to surgery and minimize or eliminate the need for general anesthetics and opioids.[28-32] They are, therefore, functionally identical from the perspective of reducing risk of cancer recurrence. Clinicians nonetheless often have a personal preference — and therefore greater experience and comfort — with one technique than the other. Consequently, we will allow either method. While perhaps sounding unlikely, previous large studies have randomized patients to regional *versus* general anesthesia to evaluate other outcomes. We thus do not anticipate difficulty finding a suitable number of consenting patients. And in fact, we have not so far had unusual difficulty enrolling more than 100 patients.

Paravertebral anesthesia can be performed with or without a catheter, and there are advantages and disadvantages to each. For example, the multi-level approach is technically easier, provides better intraoperative coverage, can be used bilaterally, and is probably safer. On the other hand, postoperative analgesia usually lasts only until the first postoperative morning whereas analgesia can be continued as long as necessary with a catheter. We note, though, that many patients (by far the majority at the Cleveland Clinic) are discharged on the first postoperative day; furthermore, the first postoperative day and evening is the period during which most opioids are required. These patients would benefit little from a catheter since it would be removed before discharge in any case. Accordingly, we will leave the choice of paravertebral technique to the

discretion of the attending anesthesiologist who will best be able to judge the relative merits of each approach in particular patients. Either technique will well serve our goal of minimizing or eliminating surgical stress response and the need for postoperative opioids.

In most patients having paravertebral or epidural anesthesia, the regional block will be combined with propofol sedation. However, not all patients tolerate regional anesthesia alone. A "light" propofol-based general anesthesia will be permitted when clinically indicated. Since propofol has little or no effect on tumor cells *in vitro*[79] or *in vivo*,[93] it is unlikely that either approach will prevent the putative benefits of regional anesthesia on surgical stress and opioid requirement. We note that all patients in our preliminary study, in which a large benefit of regional analgesia was demonstrated, received general anesthesia with sevoflurane. It is unlikely that light general anesthesia in a fraction of the patients assigned to regional anesthesia will much influence cancer recurrence.

We will give fentanyl during induction of regional or general anesthesia because doing so limits hemodynamic responses and makes induction safer. However, the small dose intravenous fentanyl we propose has a short context-sensitive half life so plasma concentrations will approach zero within 15 minutes.[221]

As mentioned in the Background section, the relative central and peripheral contributions to opioid-induced impairment of cell-mediated immunity remain controversial. While the "cleanest" approach would be to avoid combining opioids with local anesthetic infusions, these drugs usually are combined in clinical practice because adding tiny-dose opioid permits use of a lower anesthetic concentration with improved analgesia. Furthermore, fentanyl — the drug usually used with local anesthetics — is highly soluble and thus poorly transmitted by cerebrospinal fluid. And finally, only subcutaneous opioid impaired cell-mediated immunity when equi-analgesic doses of subcutaneous and intrathecal morphine were injected.[106] It is therefore unlikely that much epidural opioid will reach the peri-aqueductal gray area where opioid-induced immune suppression seems centered.[105]

Intravenous postoperative analgesia will be restricted to morphine (if tolerated), although other opioids could be used (i.e., fentanyl patch). However, there is little reason to believe morphine differs markedly from other μ-receptor opioids in regards to immune suppression and release of tumor-promoting factors.[21,97] Restricting analgesia to a single opioid to the extent possible will facilitate comparison of doses in each treatment group. When clinically necessary, other opioids will be permitted and converted to "morphine equivalents" for analysis purposes.

The major factors determining cancer recurrence risk are tumor histology, size, margins, estrogen-receptor status, and the number of positive nodes. Most will be unknown at the time of surgery, making it difficult to use a stratified randomization. Furthermore, stratification is unnecessary in large trials.[222] For example, we stratified only by study site in all the trials listed in the appendix. It is highly unlikely that stratification for any recurrence risk factor would add value to the large trials we plan, and therefore propose a randomization stratified only by study site.

A more serious issue is that we will not attempt to standardize postoperative chemotherapy or radiation, although both influence recurrence risk. For example, women who undergo breast conservation surgery without radiation therapy have a higher rate of local recurrence compared to those who receive radiation therapy: 39 versus 14 percent in one study with twenty-year follow-up.[223] Although we would obviously prefer to control such important confounding factors, we do not believe that it will be practical since both are highly influenced by patient preference and national norms. However, there is no reason to believe that use of chemotherapy or radiation will be anything but randomly distributed in the large study we propose. Furthermore, our statistical plan includes a multivariable analysis that will normalize all major potential confounding factors; although the results of this analysis will not be the primary outcome for the study, the univariable analysis will provide additional important information on our understanding of the relative efficacy of regional versus general anesthesia.

Patients will not be specifically told to which group they were assigned, but most will recognize which type of analgesia they received. It seems highly unlikely, though, that this information will influence cancer recurrence rates, especially as participants will only be told that we are testing two techniques rather than the

specific hypothesis that regional analgesia reduces risk. Similarly, it will not be possible to blind the perioperative personnel or the surgeons. However, all post-discharge evaluations will be performed by investigators who are fully blinded to randomization and actual treatment received. Contact with study patients will be highly scripted by our social worker and will avoid questions that might unbind the studies. In the event that one follow-up investigator is inadvertently unblinded to a particular patient's treatment, another blinded investigator will be substituted.

Opioids impair numerous immune functions, including those most important for containing tumors. However, opioids appear beneficial in certain circumstances. For example, withdrawal from morphine in dependent mice is immunosuppressive.[224] Furthermore, low analgesic perioperative doses of morphine have a favorable immunomodulatory effect on surgical stress and metastasis promotion[225-227] *compared to no analgesia*. These studies simply show that opioids are preferable to unrelieved stress of opioid withdrawal or untreated surgical pain in animals. However, humans are always given postoperative analgesia; our question is whether opioids are worse than other analgesic techniques such as regional analgesia. Existing data and our preliminary results suggest that they are.

That surgery provokes a neuroendocrine stress response that impairs immune function, including cell-mediated immunity, is well established in animals and humans. Similarly, that general anesthetics and opioids impair cell-mediated immunity in animals and humans is well established. Additional studies of stress mediators or cellular immunity will not answer the clinical question we ask which is whether blocking these known pathways with regional anesthesia and analgesia reduces the *clinical* risk of cancer recurrence. We thus do not propose to evaluate measures of stress and immune function.

We recognize that some participating patients will require re-operation either for control of their cancers or for unrelated reasons. Most will occur outside our control and without our knowledge. We will thus make no attempt to control anesthetic management for additional operations. However, we will record the nature of the surgery and the type of anesthesia used. These factors will be included in our multi-variate analysis.

Significance

One woman in eight develops breast cancer. Effective treatment hinges on surgical removal of the primary tumor, but surgery is usually associated with release of tumor cells into the blood stream or lymphatic system, and pre-existing scattered micrometastases most commonly remain. Whether this minimal residual disease succeeds in establishing itself as recurrence or metastases is a function of host defense and other aspects of the physiological milieu. In practice, the immune system frequently fails to neutralize remaining malignant tissue; consequently, one patient in three develops postoperative metastatic disease — and breast cancer remains the second leading cause of cancer death in women.

Considerable *in vitro* data and *in vivo* animal studies suggest that three factors associated with cancer surgery impair cellular immunity (*i.e.*, natural killer cell function): stress response to tissue injury, general anesthesia, and opioid analgesia. Our preliminary results support this possibility: the breast cancer recurrence rate was reduced by a factor-of-four ($P = 0.01$) in patients given regional analgesia rather than post-operative morphine. Results were similar in patients given epidural or opioid analgesia for prostate cancer.

Confirming our hypothesis that regional anesthesia & analgesia for breast cancer surgery reduces the risk of recurrence would be immediately applicable since regional blocks are routine procedures that are familiar to most anesthesiologists. Our study has the potential to convincingly demonstrate that a small modification to anesthetic management — one that can be implemented with little risk or cost — will reduce the risk of metastases, a complication that is ultimately lethal in most cases.

E. Human Subjects Risks and Protection

The proposed trial has already been approved by the Cleveland Clinic Human Subjects Protection Program and the IRB at five of the seven participating institutions. Written informed consent will be obtained from each participating patient. Patients who decline to participate will be given regional or general anesthesia and analgesia per their preference and that of their attending anesthesiologist; research data will not be collected from these patients.

E1. Human Subjects Involvement and Characteristics:

1,100 patients will be required to provide a 85% power to detect a 30% treatment effect at an alpha level of 0.05. Please see the section titled "Sample-size Calculations and Sequential Monitoring" for additional detail.

A Data and Safety Monitoring Board (DSMB), which will not otherwise be involved in day-to-day aspects of the protocol, will evaluate all results from the proposed trial. This committee, along with the IRB, will have exclusive authority to stop the studies either because the hypotheses have been confirmed or denied or because adverse events are detected. It will be the responsibility of this committee to alert the IRB at each participating institution to any untoward toxicity in one of the study groups.

E2. Sources of Material

Data obtained routinely for clinical purposes will be used in this study. Such data include 1) anesthetic dose and safety monitoring; 2) fluid balance and transfusion requirement; 3) post-operative nausea and vomiting; 4) details of surgery and tumor histology and spread; and 5) morphometric and demographic information.

Most importantly, we will contact patients at six-month intervals to determine whether they have experienced a recurrence of their cancer. When a recurrence is reported, we will also contact their local physician to determine details of the recurrence. If the patient is unavailable, we will contact one of at least two designated "contact people" — normally one of these will be a family member — to determine whether the patient has died. If so, we will again contact health providers to determine the details including whether the death was cancer-related.

E3. Potential risks to Subjects

The primary risk of our study is randomized assignment to regional anesthesia and analgesia or to general anesthesia and morphine analgesia. Regional and general anesthesia have different risks; however, there is no evidence or consensus that either is intrinsically safer or preferable for breast surgery. Both techniques are used routinely at the preference of patients and their anesthesiologists and surgeons.

The most common risks of general anesthesia are dental damage (≈1%), post-operative nausea and vomiting (30%), and a "hung-over" feeling lasting a day or two (>50%). The most severe complications associated with general anesthesia are aspiration pneumonia, peripheral nerve damage (usually attributed to positioning problems), and brain damage or death, which usually result from airway disasters. Fortunately, these serious complications are rare (<1%).

Inserting epidural or paravertebral needles can cause a small amount of discomfort. However, patients will be given midazolam and fentanyl, generous amounts of local anesthesia, and propofol, if necessary, during blocks. Most patients don't remember the experience at all, and those that do rarely recall it as being uncomfortable. Infection associated with regional anesthesia and catheters is a theoretical risk, but extremely rare (<1%) when catheters are removed before 48 hours.

Epidural or paravertebral blocks can also cause inadvertent "high" spinal anesthesia (<1%). This complication is easily manageable, but usually requires airway control and conversion to general anesthesia. About 5% of the attempted epidural or paravertebral anesthetics will fail and need to be converted to general anesthesia; some of these patients will nonetheless have adequate postoperative analgesia, but others will require patient-controlled analgesia.

Epidural anesthesia can cause epidural hematomas that compress the spinal cord. However, hematomas are exceedingly rare complication (<1%) because a history of coagulopathy or anticoagulant medications is a contraindication to these blocks. Epidural hematomas are treatable and usually resolve

without neurological damage, but treatment is usually surgical. Paravertebral anesthesia can cause a pneumothorax; however, this complication is rare. Treatment sometimes requires insertion of a chest drainage tube, although most resolve spontaneously.

Patients and/or family members may be bothered by questions about cancer recurrence and death. However, discomfort will be reduced by restricting contact to investigators who have been trained in this process. Follow-up interactions will follow specific scripts.

Patient privacy will be fully respected. Site investigators will discuss with the subjects and their assigned representatives, the IRB-approved informed consent explaining all procedures and potential risks and the HIPAA regulations regarding privacy of their medical information. Participating patients will complete the appropriate HIPAA forms.

Confidentiality will be maintained *per* institutional procedures. Access to medical records is limited to the investigative staff. Data will be managed by study number and analyzed anonymously. All reports will be of a summary nature, and no individual will be identified. The investigators and study coordinators are keenly aware of the increasing ethical concerns of using medical information in research and its potential misuse in clinical care. They will insure that data will be handled appropriately and that confidentiality is strictly maintained. All members of the study staff will sign agreements of confidentiality.

E4. Recruitment and Informed Consent

Patients will be recruited from the Operating Room schedules. A member of the study staff will approach potential subjects in the Preoperative Clinic or surgeon's office and give a general description of the study and its purpose to the patient. Patients will not be initially recruited on the day of surgery. Patients who orally agree will be given the Informed Consent document and asked to read it carefully. If for some reason they cannot read it themselves, it will be read to them. Any questions will be fully answered. Once patients completely understand the procedure, benefits, and risks involved, they will be asked to sign and date the consent form.

Two copies of the Informed Consent form will be made. One copy remains with the patient's chart, another copy will be given to the patient, and the original will be filed in the case report form. The original consent will thus be available to the investigators, their staff, study monitors, and each institution's IRB.

E5. Protection Against Risks

The Executive Committee will have responsibility for evaluating all major morbidity occurring in study patients, and be authorized to stop the studies if excessive morbidity in one group appears related to study interventions. Additionally, any morbidity potentially related to the protocol will be reported to the IRB/Ethics Committees at each participating institution; these Committees also can stop the studies at any time for any reason.

E6. Collaborating Sites

The studies will be approved by the IRB at each of the participating sites (most already have approval). Each site will provide written confirmation that the four criteria specified by the Public Health Service for Protection of Human Subjects have been addressed.

E7. Risk-Benefit to Subjects and Importance of Knowledge to be Gained

Neuraxial anesthesia (epidural or spinal) has been best studied for hip arthroplasty. Neuraxial anesthesia reduces blood loss during hip arthroplasty[228] and usually provides better postoperative pain relief than the combination of general anesthesia and postoperative opioids. However, epidural anesthesia does not reduce mortality or the risk of myocardial infarction after hip arthroplasty, even in high risk populations.[229] Perhaps consequently, general anesthesia and opioid analgesia remain common even for hip arthroplasty and is certainly used far more often than regional anesthesia/analgesia for nearly all other operations. And as mentioned above, there is neither evidence nor consensus that any particular anesthetic or analgesic technique is preferable for breast surgery; a variety of techniques are therefore used at the preference of patients, anesthesiologists, and surgeons.

The major risk of cancer surgery is neither the surgery *per se* nor the anesthesia: it is recurrence of cancer — which is usually a lethal event. Our studies are powered to detect a reduction in recurrence risk of

30%, which is similar to that provided by postoperative radiation or chemotherapy. But switching from general anesthesia and opioid analgesia to regional anesthesia and analgesia is clearly safer and less expensive than radiation or chemotherapy. Confirming our primary hypothesis, that a straightforward change in anesthetic management reduces the risk of breast cancer recurrence, would therefore be a major advance.

E8. Gender and Minority Inclusion

Breast cancer is largely a disease of women. It is unlikely that more than a few cases in men would be available during the entire study period. Furthermore, the occasional man who gets breast cancer usually has an extremely aggressive form of the disease. We will, therefore, restrict the study to women.

We have no reason to believe that the effects of general anesthesia or morphine on immune function and release of tumor-promoting factors is substantively related to race or ethnicity. We will consequently include patients of all races and ethnicities and make a special effort to include minorities. We assume that our study population will reflect the patient population of each participating health system. In Cleveland, our participants will be roughly 10% Hispanic and 15% African American. At the other sites, there will be fewer minorities, but *minorities will be specifically solicited for enrollment at all sites to provide good ethnic/racial balance*.

Cancer may be diagnosed at later stages in minorities because they often have limited access to health care; however, minority participants will be roughly equally distributed into the two treatment groups. Our multivariable analysis will include race and ethnicity as categorical variables to adjust for an (unlikely) effect of either factor on our primary outcome.

E9. Inclusion of Children

Breast cancer is rare in children. Children will, therefore, be excluded from the proposed studies.

F. Vertebrate Animals

Not applicable.

G. Select Agent Research

Not applicable.

H. Literature Cited

1. Eschwege P, Dumas F, Blanchet P, Le Maire V, Benoit G, Jardin A, Lacour B, Loric S: Haematogenous dissemination of prostatic epithelial cells during radical prostatectomy. Lancet 1995; 346: 1528-30
2. Foss OP, Brennhovd IO, Messelt OT, Efskind J, Liverud K: Invasion of tumor cells into the bloodstream caused by palpation or biopsy of the tumor. Surgery 1966; 59: 691-5
3. Denis MG, Lipart C, Leborgne J, LeHur PA, Galmiche JP, Denis M, Ruud E, Truchaud A, Lustenberger P: Detection of disseminated tumor cells in peripheral blood of colorectal cancer patients. Int J Cancer 1997; 74: 540-4
4. Holmgren L, O'Reilly MS, Folkman J: Dormancy of micrometastases: balanced proliferation and apoptosis in the presence of angiogenesis suppression. Nat Med 1995; 1: 149-53
5. Shakhar G, Ben-Eliyahu S: Potential prophylactic measures against postoperative immunosuppression: could they reduce recurrence rates in oncological patients? Ann Surg Oncol 2003; 10: 972-92
6. Smyth MJ, Godfrey DI, Trapani JA: A fresh look at tumor immunosurveillance and immunotherapy. Nat Immunol 2001; 2: 293-9.
7. Bar-Yosef S, Melamed R, Page GG, Shakhar G, Shakhar K, Ben-Eliyahu S: Attenuation of the tumor-promoting effect of surgery by spinal blockade in rats. Anesthesiology 2001; 94: 1066-73
8. Ben-Eliyahu S, Page GG, Yirmiya R, Shakhar G: Evidence that stress and surgical interventions promote tumor development by suppressing natural killer cell activity. Int J Cancer 1999; 80: 880-8
9. Page GG, Blakely WP, Ben-Eliyahu S: Evidence that postoperative pain is a mediator of the tumor-promoting effects of surgery in rats. Pain 2001; 90: 191-9
10. Wong IH, Lau WY, Leung T, Yeo W, Johnson PJ: Hematogenous dissemination of hepatocytes and tumor cells after surgical resection of hepatocellular carcinoma: a quantitative analysis. Clin Cancer Res 1999; 5: 4021-7

11. Zetter BR: Angiogenesis and tumor metastasis. Annu Rev Med 1998; 49: 407-24
12. O'Reilly MS, Holmgren L, Shing Y, Chen C, Rosenthal RA, Moses M, Lane WS, Cao Y, Sage EH, Folkman J: Angiostatin: a novel angiogenesis inhibitor that mediates the suppression of metastases by a Lewis lung carcinoma. Cell 1994; 79: 315-28
13. O'Reilly MS, Boehm T, Shing Y, Fukai N, Vasios G, Lane WS, Flynn E, Birkhead JR, Olsen BR, Folkman J: Endostatin: an endogenous inhibitor of angiogenesis and tumor growth. Cell 1997; 88: 277-85
14. Folkman J: Tumor Angiogenesis, The Molecular Basis of Cancer. Edited by J Medelsohn PMH, M A Israel, L A Liotta, W B Saunders, 1995
15. Lutgendorf SK, Cole S, Costanzo E, Bradley S, Coffin J, Jabbari S, Rainwater K, Ritchie JM, Yang M, Sood AK: Stress-related mediators stimulate vascular endothelial growth factor secretion by two ovarian cancer cell lines. Clin Cancer Res 2003; 9: 4514-21
16. Antoni MH, Lutgendorf SK, Cole SW, Dhabhar FS, Sephton SE, McDonald PG, Stefanek M, Sood AK: The influence of bio-behavioural factors on tumour biology: pathways and mechanisms. Nat Rev Cancer 2006; 6: 240-8
17. Sacerdote P, Bianchi M, Gaspani L, Manfredi B, Maucione A, Terno G, Ammatuna M, Panerai AE: The effects of tramadol and morphine on immune responses and pain after surgery in cancer patients. Anesth Analg 2000; 90: 1411-4
18. Brand JM, Kirchner H, Poppe C, Schmucker P: The effects of general anesthesia on human peripheral immune cell distribution and cytokine production. Clin Immunol Immunopathol 1997; 83: 190-4
19. Markovic SN, Knight PR, Murasko DM: Inhibition of interferon stimulation of natural killer cell activity in mice anesthetized with halothane or isoflurane. Anesthesiology 1993; 78: 700-6
20. Shapiro J, Jersky J, Katzav S, Feldman M, Segal S: Anesthetic drugs accelerate the progression of postoperative metastases of mouse tumors. J Clin Invest 1981; 68: 678-85.
21. Beilin B, Shavit Y, Hart J, Mordashov B, Cohn S, Notti I, Bessler H: Effects of anesthesia based on large versus small doses of fentanyl on natural killer cell cytotoxicity in the perioperative period. Anesth Analg 1996; 82: 492-7
22. Yeager MP, Colacchio TA, Yu CT, Hildebrandt L, Howell AL, Weiss J, Guyre PM: Morphine inhibits spontaneous and cytokine-enhanced natural killer cell cytotoxicity in volunteers. Anesthesiology 1995; 83: 500-8
23. Gupta K, Kshirsagar S, Chang L, Schwartz R, Law PY, Yee D, Hebbel RP: Morphine stimulates angiogenesis by activating proangiogenic and survival-promoting signaling and promotes breast tumor growth. Cancer Res 2002; 62: 4491-8
24. Kehlet H: The stress response to surgery, Neural blockade in anaesthesia and pain management. Edited by Cousins M, Brinderbough M. New York, Lippincott-Williams, 1998
25. Buggy DJ, Smith G: Epidural anaesthesia and analgesia: better outcome after major surgery?. Growing evidence suggests so. Bmj 1999; 319: 530-1
26. O'Riain SC, Buggy DJ, Kerin MJ, Watson RW, Moriarty DC: Inhibition of the stress response to breast cancer surgery by regional anesthesia and analgesia does not affect vascular endothelial growth factor and prostaglandin E2. Anesth Analg 2005; 100: 244-9
27. Chae BK, Lee HW, Sun K, Choi YH, Kim HM: The effect of combined epidural and light general anesthesia on stress hormones in open heart surgery patients. Surg Today 1998; 28: 727-31
28. Najarian MM, Johnson JM, Landercasper J, Havlik P, Lambert PJ, McCarthy D: Paravertebral block: an alternative to general anesthesia in breast cancer surgery. Am Surg 2003; 69: 213-8; discussion 8
29. Greengrass R, O'Brien F, Lyerly K, Hardman D, Gleason D, D'Ercole F, Steele S: Paravertebral block for breast cancer surgery. Can J Anaesth 1996; 43: 858-61
30. Weltz CR, Greengrass RA, Lyerly HK: Ambulatory surgical management of breast carcinoma using paravertebral block. Ann Surg 1995; 222: 19-26
31. Coveney E, Weltz CR, Greengrass R, Iglehart JD, Leight GS, Steele SM, Lyerly HK: Use of paravertebral block anesthesia in the surgical management of breast cancer: experience in 156 cases. Ann Surg 1998; 227: 496-501
32. Kairaluoma PM, Bachmann MS, Korpinen AK, Rosenberg PH, Pere PJ: Single-injection paravertebral block before general anesthesia enhances analgesia after breast cancer surgery with and without associated lymph node biopsy. Anesth Analg 2004; 99: 1837-43
33. American Cancer Society: Cancer facts and figures. 2005

34. Wittekind C, Neid M: Cancer invasion and metastasis. Oncology 2005; 69 Suppl 1: 14-6
35. Nagrath S, Sequist LV, Maheswaran S, Bell DW, Irimia D, Ulkus L, Smith MR, Kwak EL, Digumarthy S, Muzikansky A, Ryan P, Balis UJ, Tompkins RG, Haber DA, Toner M: Isolation of rare circulating tumour cells in cancer patients by microchip technology. Nature 2007; 450: 1235-9
36. Fidler IJ: The pathogenesis of cancer metastasis: the 'seed and soil' hypothesis revisited. Nat Rev Cancer 2003; 3: 453-8
37. Anand-Apte B, Fox PL, Borden EC: Tumor angiogenesis, Current clinical oncology, melanoma: biologicaly targeted therapeutics. Totowa, Humana Press, 2002, pp 325-60
38. Kaplan RN, Riba RD, Zacharoulis S, Bramley AH, Vincent L, Costa C, MacDonald DD, Jin DK, Shido K, Kerns SA, Zhu Z, Hicklin D, Wu Y, Port JL, Altorki N, Port ER, Ruggero D, Shmelkov SV, Jensen KK, Rafii S, Lyden D: VEGFR1-positive haematopoietic bone marrow progenitors initiate the pre-metastatic niche. Nature 2005; 438: 820-7
39. O'Reilly MS, Holmgren L, Shing Y, Chen C, Rosenthal RA, Moses M, Lane WS, Cao Y, Sage EH, Folkman J: Angiostatin: a novel angiogenesis inhibitor that mediates the suppression of metastases by a Lewis lung carcinoma. Cell 1994; 79: 315-28
40. O'Reilly MS, Boehm T, Shing Y, Fukai N, Vasios G, Lane WS, Flynn E, Birkhead JR, Olsen BR, Folkman J: Endostatin: an endogenous inhibitor of angiogenesis and tumor growth. Cell 1997; 88: 277-85
41. Hewitt HB: Second point: animal tumor models and their relevance to human tumor immunology. J Biol Response Mod 1983; 2: 210-6
42. Killion JJ, Fidler IJ: Therapy of cancer metastasis by tumoricidal activation of tissue macrophages using liposome-encapsulated immunomodulators. Pharmacol Ther 1998; 78: 141-54
43. McCoy JL, Rucker R, Petros JA: Cell-mediated immunity to tumor-associated antigens is a better predictor of survival in early stage breast cancer than stage, grade or lymph node status. Breast Cancer Res Treat 2000; 60: 227-34
44. Taketomi A, Shimada M, Shirabe K, Kajiyama K, Gion T, Sugimachi K: Natural killer cell activity in patients with hepatocellular carcinoma: a new prognostic indicator after hepatectomy. Cancer 1998; 83: 58-63
45. Mansi JL, Gogas H, Bliss JM, Gazet JC, Berger U, Coombes RC: Outcome of primary-breast-cancer patients with micrometastases: a long-term follow-up study. Lancet 1999; 354: 197-202
46. Dixon M: ABC of Breast Diseases, 2nd Edition. London, BMJ Publishing, 2000
47. Da Costa ML, Redmond P, Bouchier-Hayes DJ: The effect of laparotomy and laparoscopy on the establishment of spontaneous tumor metastases. Surgery 1998; 124: 516-25
48. Yamaguchi K, Takagi Y, Aoki S, Futamura M, Saji S: Significant detection of circulating cancer cells in the blood by reverse transcriptase-polymerase chain reaction during colorectal cancer resection. Ann Surg 2000; 232: 58-65
49. Tonnesen E: Immunological aspects of anaesthesia and surgery--with special reference to NK cells. Dan Med Bull 1989; 36: 263-81
50. Zoller M, Heumann U, Betzler M, Stimmel H, Matzku S: Depression of nonadaptive immunity after surgical stress: influence on metastatic spread. Invasion Metastasis 1989; 9: 46-68
51. Shakhar K, Shakhar G, Rosenne E, Ben-Eliyahu S: Timing within the menstrual cycle, sex, and the use of oral contraceptives determine adrenergic suppression of NK cell activity. Br J Cancer 2000; 83: 1630-6
52. Abramovitch R, Marikovsky M, Meir G, Neeman M: Stimulation of tumour growth by wound-derived growth factors. Br J Cancer 1999; 79: 1392-8
53. Hofer SO, Molema G, Hermens RA, Wanebo HJ, Reichner JS, Hoekstra HJ: The effect of surgical wounding on tumour development. Eur J Surg Oncol 1999; 25: 231-43
54. Vallejo R, Hord ED, Barna SA, Santiago-Palma J, Ahmed S: Perioperative immunosuppression in cancer patients. J Environ Pathol Toxicol Oncol 2003; 22: 139-46
55. Madden KS, Sanders VM, Felten DL: Catecholamine influences and sympathetic neural modulation of immune responsiveness. Annu Rev Pharmacol Toxicol 1995; 35: 417-48
56. Pirttikangas CO, Salo M, Riutta A, Perttila J, Peltola O, Kirvela O: Effects of propofol and Intralipid on immune response and prostaglandin E2 production. Anaesthesia 1995; 50: 317-21
57. Rossano F, Tufano R, Cipollaro de LEG, Servillo G, Baroni A, Tufano MA: Anesthetic agents induce human mononuclear leucocytes to release cytokines. Immunopharmacol Immunotoxicol 1992; 14: 439-50

58. Galley HF, Dubbels AM, Webster NR: The effect of midazolam and propofol on interleukin-8 from human polymorphonuclear leukocytes. Anesth Analg 1998; 86: 1289-93
59. Sato W, Enzan K, Masaki Y, Kayaba M, Suzuki M: [The effect of isoflurane on the secretion of TNF-alpha and IL-1 beta from LPS-stimulated human peripheral blood monocytes]. Masui 1995; 44: 971-5
60. Herberman RB, Ortaldo JR: Natural killer cells: their roles in defenses against disease. Science 1981; 214: 24-30
61. Talmadge JE, Meyers KM, Prieur DJ, Starkey JR: Role of NK cells in tumour growth and metastasis in beige mice. Nature 1980; 284: 622-4
62. Page GG: Surgery-induced immunosuppression and postoperative pain management. AACN Clin Issues 2005; 16: 302-9; quiz 416-8
63. Munford RS, Pugin J: Normal responses to injury prevent systemic inflammation and can be immunosuppressive. Am J Respir Crit Care Med 2001; 163: 316-21
64. Page GG, Ben-Eliyahu S: Indomethacin attenuates the immunosuppressive and tumor-promoting effects of surgery. J Pain 2002; 3: 301-8
65. Ben-Eliyahu S: The promotion of tumor metastasis by surgery and stress: immunological basis and implications for psychoneuroimmunology. Brain Behav Immun 2003; 17 Suppl 1: S27-36
66. Melamed R, Rosenne E, Shakhar K, Schwartz Y, Abudarham N, Ben-Eliyahu S: Marginating pulmonary-NK activity and resistance to experimental tumor metastasis: suppression by surgery and the prophylactic use of a beta-adrenergic antagonist and a prostaglandin synthesis inhibitor. Brain Behav Immun 2005; 19: 114-26
67. Shankar N, Varshney A, Bhattacharya A, Sharma KN: Electroacupuncture, morphine and clonidine: a comparative study of analgesic effects. Indian Journal of Physiology & Pharmacology 1996; 40: 225-30
68. Welters ID, Menzebach A, Goumon Y, Langefeld TW, Teschemacher H, Hempelmann G, Stefano GB: Morphine suppresses complement receptor expression, phagocytosis, and respiratory burst in neutrophils by a nitric oxide and mu(3) opiate receptor-dependent mechanism. J Neuroimmunol 2000; 111: 139-45
69. Welters ID, Menzebach A, Goumon Y, Cadet P, Menges T, Hughes TK, Hempelmann G, Stefano GB: Morphine inhibits NF-kappaB nuclear binding in human neutrophils and monocytes by a nitric oxide-dependent mechanism. Anesthesiology 2000; 92: 1677-84
70. Xu YX, Ayala A, Chaudry IH: Prolonged immunodepression after trauma and hemorrhagic shock. J Trauma 1998; 44: 335-41
71. Clarke PJ, Burton RC, Wood KJ: Allogeneic blood transfusion reduces murine pulmonary natural killer (NK) activity and enhances lung metastasis of a syngeneic tumour. Int J Cancer 1993; 55: 996-1002
72. Nielsen HJ: Detrimental effects of perioperative blood transfusion. Br J Surg 1995; 82: 582-7
73. Yucel Y, Barlan M, Lenhardt R, Kurz A, Sessler DI: Perioperative hypothermia does not enhance the risk of cancer dissemination. Am J Surg 2005; 189: 651-5
74. Ben-Eliyahu S, Shakhar G, Shakhar K, Melamed R: Timing within the oestrous cycle modulates adrenergic suppression of NK activity and resistance to metastasis: possible clinical implications. Br J Cancer 2000; 83: 1747-54
75. Page GG, Ben-Eliyahu S: The immune-suppressive nature of pain. Semin Oncol Nurs 1997; 13: 10-5
76. Carr DJ, Gerak LR, France CP: Naltrexone antagonizes the analgesic and immunosuppressive effects of morphine in mice. J Pharmacol Exp Ther 1994; 269: 693-8
77. Ben-Eliyahu S, Shakhar G, Page GG, Stefanski V, Shakhar K: Suppression of NK cell activity and of resistance to metastasis by stress: a role for adrenal catecholamines and beta-adrenoceptors. Neuroimmunomodulation 2000; 8: 154-64
78. Stefanski V, Ben-Eliyahu S: Social confrontation and tumor metastasis in rats: defeat and beta-adrenergic mechanisms. Physiol Behav 1996; 60: 277-82
79. Melamed R, Bar-Yosef S, Shakhar G, Shakhar K, Ben-Eliyahu S: Suppression of natural killer cell activity and promotion of tumor metastasis by ketamine, thiopental, and halothane, but not by propofol: mediating mechanisms and prophylactic measures. Anesth Analg 2003; 97: 1331-9
80. Erskine R, Janicki PK, Ellis P, James MF: Neutrophils from patients undergoing hip surgery exhibit enhanced movement under spinal anaesthesia compared with general anaesthesia. Can J Anaesth 1992; 39: 905-10
81. Loop T, Scheiermann P, Doviakue D, Musshoff F, Humar M, Roesslein M, Hoetzel A, Schmidt R, Madea B, Geiger KK, Pahl HL, Pannen BH: Sevoflurane inhibits phorbol-myristate-acetate-induced activator protein-1

activation in human T lymphocytes in vitro: potential role of the p38-stress kinase pathway. Anesthesiology 2004; 101: 710-21
82. Lieners C, Redl H, Schlag G, Hammerschmidt DE: Inhibition by halothane, but not by isoflurane, of oxidative response to opsonized zymosan in whole blood. Inflammation 1989; 13: 621-30
83. Loop T, Dovi-Akue D, Frick M, Roesslein M, Egger L, Humar M, Hoetzel A, Schmidt R, Borner C, Pahl HL, Geiger KK, Pannen BH: Volatile anesthetics induce caspase-dependent, mitochondria-mediated apoptosis in human T lymphocytes in vitro. Anesthesiology 2005; 102: 1147-57
84. Procopio MA, Rassias AJ, DeLeo JA, Pahl J, Hildebrandt L, Yeager MP: The in vivo effects of general and epidural anesthesia on human immune function. Anesth Analg 2001; 93: 460-5, 4th contents page
85. Kripke BJ, Kupferman A, Luu KC: Suppression of chemotaxis to corneal inflammation by nitrous oxide. Zhonghua Min Guo Wei Sheng Wu Ji Mian Yi Xue Za Zhi 1987; 20: 302- 10.
86. Perry J, Chanarin I, Deacon R, Lumb M: Chronic cobalamin inactivation impairs folate polyglutamate synthesis in the rat. J Clin Invest 1983; 71: 1183-90
87. Skacel PO, Hewlett AM, Lewis JD, Lumb M, Nunn JF, Chanarin I: Studies on the haemopoetic toxicity of nitrous oxide in man. Br J Haematol 1983; 53: 693-701
88. Rowland AS, Baird DD, Shore DL, Weinberg CR, Savitz DA, Wilcox AJ: Nitrous oxide and spontaneous abortion in female dental assistants. Am J Epidemiol 1995; 141: 531-8
89. Krumholz W, Endrass J, Hempelmann G: Propofol inhibits phagocytosis and killing of Staphylococcus aureus and Escherichia coli by polymorphonuclear leukocytes in vitro. Can J Anaesth 1994; 41: 446-9
90. O'Donnell NG, McSharry CP, Wilkinson PC, Asbury AJ: Comparison of the inhibitory effect of propofol, thiopentone and midazolam on neutrophil polarization in vitro in the presence or absence of human serum albumin. Br J Anaesth 1992; 69: 70-4
91. Pirttikangas CO, Perttila J, Salo M: Propofol emulsion reduces proliferative responses of lymphocytes from intensive care patients. Intensive Care Med 1993; 19: 299-302
92. Pirttikangas CO, Salo M, Peltola O: Propofol infusion anaesthesia and the immune response in elderly patients undergoing ophthalmic surgery. Anaesthesia 1996; 51: 318-23
93. Mammoto T, Mukai M, Mammoto A, Yamanaka Y, Hayashi Y, Mashimo T, Kishi Y, Nakamura H: Intravenous anesthetic, propofol inhibits invasion of cancer cells. Cancer Lett 2002; 184: 165-70
94. McCarthy L, Wetzel M, Sliker JK, Eisenstein TK, Rogers TJ: Opioids, opioid receptors, and the immune response. Drug Alcohol Depend 2001; 62: 111-23
95. Vallejo R, de Leon-Casasola O, Benyamin R: Opioid therapy and immunosuppression: a review. Am J Ther 2004; 11: 354-65
96. Eisenstein TK, Rogers TJ, Meissler JJ, Jr., Adler MW, Hilburger ME: Morphine depresses macrophage numbers and function in mouse spleens. Adv Exp Med Biol 1998; 437: 33-41
97. Shavit Y, Ben-Eliyahu S, Zeidel A, Beilin B: Effects of fentanyl on natural killer cell activity and on resistance to tumor metastasis in rats. Dose and timing study. Neuroimmunomodulation 2004; 11: 255-60
98. Rahim RT, Meissler JJ, Jr., Cowan A, Rogers TJ, Geller EB, Gaughan J, Adler MW, Eisenstein TK: Administration of mu-, kappa- or delta2-receptor agonists via osmotic minipumps suppresses murine splenic antibody responses. Int Immunopharmacol 2001; 1: 2001-9
99. Wei G, Moss J, Yuan CS: Opioid-induced immunosuppression: is it centrally mediated or peripherally mediated? Biochem Pharmacol 2003; 65: 1761-6
100. Stefano GB, Burrill JD, Labur S, Blake J, Cadet P: Regulation of various genes in human leukocytes acutely exposed to morphine: expression microarray analysis. Med Sci Monit 2005; 11: MS35-MS42
101. Eisenstein TK, Hilburger ME: Opioid modulation of immune responses: effects on phagocyte and lymphoid cell populations. Journal of Neuroimmunology 1998; 83: 36-44
102. Hall DM, Suo JL, Weber RJ: Opioid mediated effects on the immune system: sympathetic nervous system involvement. J Neuroimmunol 1998; 83: 29-35
103. Rahim RT, Meissler JJ, Jr., Adler MW, Eisenstein TK: Splenic macrophages and B cells mediate immunosuppression following abrupt withdrawal from morphine. J Leukoc Biol 2005; 78: 1185-91
104. Shavit Y, Depaulis A, Martin FC, Terman GW, Pechnick RN, Zane CJ, Gale RP, Liebeskind JC: Involvement of brain opiate receptors in the immune-suppressive effect of morphine. Proc Natl Acad Sci U S A 1986; 83: 7114-7

105. Hernandez MC, Flores LR, Bayer BM: Immunosuppression by morphine is mediated by central pathways. J Pharmacol Exp Ther 1993; 267: 1336-41
106. Hamra JG, Yaksh TL: Equianalgesic doses of subcutaneous but not intrathecal morphine alter phenotypic expression of cell surface markers and mitogen-induced proliferation in rat lymphocytes. Anesthesiology 1996; 85: 355-65
107. Stefano GB, Hartman A, Bilfinger TV, Magazine HI, Liu Y, Casares F, Goligorsky MS: Presence of the mu3 opiate receptor in endothelial cells Coupling to nitric oxide production and vasodilation. J Biol Chem 1995; 270: 30290-3
108. Fimiani C, Mattocks D, Cavani F, Salzet M, Deutsch DG, Pryor S, Bilfinger TV, Stefano GB: Morphine and anandamide stimulate intracellular calcium transients in human arterial endothelial cells: coupling to nitric oxide release. Cell Signal 1999; 11: 189-93
109. Lee PC, Salyapongse AN, Bragdon GA, Shears LL, Watkins SC, Edington HD, Billiar TR: Impaired wound healing and angiogenesis in eNOS-deficient mice. Am J Physiol 1999; 277: H1600-H8
110. Pasi A, Qu BX, Steiner R, Senn HJ, Bar W, Messiha FS: Angiogenesis: modulation with opioids. Gen Pharmacol 1991; 22: 1077-9
111. Poonawala T, Levay-Young BK, Hebbel RP, Gupta K: Opioids heal ischemic wounds in the rat. Wound Repair and Regeneration 2005; 13: 165-74
112. Balasubramanian S, Ramakrishnan S, Charboneau R, Wang J, Barke RA, Roy S: Morphine sulfate inhibits hypoxia-induced vascular endothelial growth factor expression in endothelial cells and cardiac myocytes. J Mol Cell Cardiol 2001; 33: 2179-87
113. Kendall RL, Thomas KA: Inhibition of Vascular Endothelial Cell Growth Factor Activity by an Endogeneously Encoded Soluble Receptor. Proceedings of the National Academy of Sciences 1993; 90: 10705-9
114. Kendall RL, Wang G, Thomas KA: Identification of a natural soluble form of the vascular endothelial growth factor receptor, FLT-1, and its heterodimerization with KDR. Biochem Biophys Res Commun 1996; 226: 324-8
115. Roeckl W, Hecht D, Sztajer H, Waltenberger J, Yayon A, Weich HA: Differential binding characteristics and cellular inhibition by soluble VEGF receptors 1 and 2. Exp Cell Res 1998; 241: 161-70
116. Hoar FJ, Lip GY, Belgore F, Stonelake PS: Circulating levels of VEGF-A, VEGF-D and soluble VEGF-A receptor (sFIt-1) in human breast cancer. Int J Biol Markers 2004; 19: 229-35
117. Singleton PA, Lingen MW, Fekete MJ, Garcia JGN, Moss J: Methylnaltrexone inhibits opiate and VEGF-induced angiogenesis: Role of receptor transactivation. Microvasc Res, in press
118. Takeuchi I, Ishida H, Mori T, Hashimoto D: Comparison of the effects of gasless procedure, CO2-pneumoperitoneum, and laparotomy on splenic and hepatic natural killer activity in a rat model. Surg Endosc 2004; 18: 255-60
119. Ben-Eliyahu S: The price of anticancer intervention. Does surgery promote metastasis? Lancet Oncol 2002; 3: 578-9
120. Caballero-Hernandez D, Weber RJ, Hicks ME, Tamez-Guerra R, Rodriguez-Padilla C, Tamez-Guerra P, Rice KC, Ananthan S, Gomez-Flores R: Potentiation of rat lymphocyte proliferation by novel non-peptidic synthetic opioids. Int Immunopharmacol 2005; 5: 1271-8
121. Taguchi A, Sharma N, Saleem RM, Sessler DI, Carpenter RL, Seyedsadr M, Kurz A: Selective postoperative inhibition of gastrointestinal opioid receptors. N Engl J Med 2001; 345: 935-40.
122. Yokoyama M, Itano Y, Katayama H, Morimatsu H, Takeda Y, Takahashi T, Nagano O, Morita K: The effects of continuous epidural anesthesia and analgesia on stress response and immune function in patients undergoing radical esophagectomy. Anesth Analg 2005; 101: 1521-7
123. Volk T, Schenk M, Voigt K, Tohtz S, Putzier M, Kox WJ: Postoperative epidural anesthesia preserves lymphocyte, but not monocyte, immune function after major spine surgery. Anesth Analg 2004; 98: 1086-92
124. Pflug AE, Halter JB: Effect of spinal anesthesia on adrenergic tone and the neuroendocrine responses to surgical stress in humans. Anesthesiology 1981; 55: 120-6
125. Koltun WA, Bloomer MM, Tilberg AF, Seaton JF, Ilahi O, Rung G, Gifford RM, Kauffman GL, Jr.: Awake epidural anesthesia is associated with improved natural killer cell cytotoxicity and a reduced stress response. Am J Surg 1996; 171: 68-72; discussion -3
126. Tonnesen E, Wahlgreen C: Influence of extradural and general anaesthesia on natural killer cell activity and lymphocyte subpopulations in patients undergoing hysterectomy. Br J Anaesth 1988; 60: 500-7

127. Ryhanen P, Jouppila R, Lanning M, Jouppila P, Hollmen A, Kouvalainen K: Natural killer cell activity after elective cesarean section under general and epidural anesthesia in healthy parturients and their newborns. Gynecol Obstet Invest 1985; 19: 139-42
128. Le Cras AE, Galley HF, Webster NR: Spinal but not general anesthesia increases the ratio of T helper 1 to T helper 2 cell subsets in patients undergoing transurethral resection of the prostate. Anesth Analg 1998; 87: 1421-5
129. Wada H, Seki S, Takahashi T, Kawarabayashi N, Higuchi H, Habu Y, Sugahara S, Kazama T: Combined spinal and general anesthesia attenuates liver metastasis by preserving TH1/TH2 cytokine balance. Anesthesiology 2007; 106: 499-506
130. Rutberg H, Hakanson E, Anderberg B, Jorfeldt L, Martensson J, Schildt B: Effects of the extradural administration of morphine, or bupivacaine, on the endocrine response to upper abdominal surgery. Br J Anaesth 1984; 56: 233-8
131. Schlagenhauff B, Ellwanger U, Breuninger H, Stroebel W, Rassner G, Garbe C: Prognostic impact of the type of anaesthesia used during the excision of primary cutaneous melanoma. Melanoma Res 2000; 10: 165-9
132. Macrae WA: Chronic pain after surgery. Br J Anaesth 2001; 87: 88-98
133. Iohom G, Abdalla H, O'Brien J, Szarvas S, Larney V, Buckley E, Butler M, Shorten GD: The associations between severity of early postoperative pain, chronic postsurgical pain and plasma concentration of stable nitric oxide products after breast surgery. Anesth Analg 2006; 103: 995-1000
134. Dajczman E, Gordon A, Kreisman H, Wolkove N: Long-term postthoracotomy pain. Chest 1991; 99: 270-4
135. Gottschalk A, Smith DS, Jobes DR, Kennedy SK, Lally SE, Noble VE, Grugan KF, Seifert HA, Cheung A, Malkowicz SB, Gutsche BB, Wein AJ: Preemptive epidural analgesia and recovery from radical prostatectomy: a randomized controlled trial. Jama 1998; 279: 1076-82
136. Haythornthwaite JA, Raja SN, Fisher B, Frank SM, Brendler CB, Shir Y: Pain and quality of life following radical retropubic prostatectomy. J Urol 1998; 160: 1761-4
137. Katz J, Cohen L: Preventive analgesia is associated with reduced pain disability 3 weeks but not 6 months after major gynecologic surgery by laparotomy. Anesthesiology 2004; 101: 169-74
138. Bruce J, Poobalan AS, Smith WC, Chambers WA: Quantitative assessment of chronic postsurgical pain using the McGill Pain Questionnaire. Clin J Pain 2004; 20: 70-5
139. Kalso E, Mennander S, Tasmuth T, Nilsson E: Chronic post-sternotomy pain. Acta Anaesthesiol Scand 2001; 45: 935-9
140. Bay-Nielsen M, Perkins FM, Kehlet H: Pain and functional impairment 1 year after inguinal herniorrhaphy: a nationwide questionnaire study. Ann Surg 2001; 233: 1-7
141. Callesen T, Kehlet H: [Inguinal herniotomy--which kind of anesthesia? Economical considerations]. Ugeskr Laeger 1995; 157: 421-4
142. Kaya FN, Turker G, Basagan-Mogol E, Goren S, Bayram S, Gebitekin C: Preoperative multiple-injection thoracic paravertebral blocks reduce postoperative pain and analgesic requirements after video-assisted thoracic surgery. J Cardiothorac Vasc Anesth 2006; 20: 639-43
143. Klein SM, Bergh A, Steele SM, Georgiade GS, Greengrass RA: Thoracic paravertebral block for breast surgery. Anesth Analg 2000; 90: 1402-5
144. Renck H: Time for revivification of paravertebral blocks? Acta Anaesthesiol Scand 1995; 39: 1003-4
145. Richardson J, Lonnqvist PA: Thoracic paravertebral block. Br J Anaesth 1998; 81: 230-8
146. Richardson J, Sabanathan S, Jones J, Shah RD, Cheema S, Mearns AJ: A prospective, randomized comparison of preoperative and continuous balanced epidural or paravertebral bupivacaine on post-thoracotomy pain, pulmonary function and stress responses. Br J Anaesth 1999; 83: 387-92
147. Teasdale C, McCrum AM, Williams NB, Horton RE: A randomised controlled trial to compare local with general anaesthesia for short-stay inguinal hernia repair. Ann R Coll Surg Engl 1982; 64: 238-42
148. Terheggen MA, Wille F, Borel Rinkes IH, Ionescu TI, Knape JT: Paravertebral blockade for minor breast surgery. Anesth Analg 2002; 94: 355-9
149. Tverskoy M, Cozacov C, Ayache M, Bradley EL, Jr., Kissin I: Postoperative pain after inguinal herniorrhaphy with different types of anesthesia. Anesth Analg 1990; 70: 29-35
150. Block BM, Liu SS, Rowlingson AJ, Cowan AR, Cowan JA, Jr., Wu CL: Efficacy of postoperative epidural analgesia: a meta-analysis. Jama 2003; 290: 2455-63

151. Obata H, Saito S, Fujita N, Fuse Y, Ishizaki K, Goto F: Epidural block with mepivacaine before surgery reduces long-term post-thoracotomy pain. Can J Anaesth 1999; 46: 1127-32
152. Senturk M, Ozcan PE, Talu GK, Kiyan E, Camci E, Ozyalcin S, Dilege S, Pembeci K: The effects of three different analgesia techniques on long-term postthoracotomy pain. Anesth Analg 2002; 94: 11-5
153. Kairaluoma PM, Bachmann MS, Rosenberg PH, Pere PJ: Preincisional paravertebral block reduces the prevalence of chronic pain after breast surgery. Anesth Analg 2006; 103: 703-8
154. Nikolajsen L, Sorensen HC, Jensen TS, Kehlet H: Chronic pain following Caesarean section. Acta Anaesthesiol Scand 2004; 48: 111-6
155. Brennan TJ, Kehlet H: Preventive analgesia to reduce wound hyperalgesia and persistent postsurgical pain: not an easy path. Anesthesiology 2005; 103: 681-3
156. Fassoulaki A, Sarantopoulos C, Melemeni A, Hogan Q: EMLA reduces acute and chronic pain after breast surgery for cancer. Reg Anesth Pain Med 2000; 25: 350-5
157. Fassoulaki A, Sarantopoulos C, Melemeni A, Hogan Q: Regional block and mexiletine: the effect on pain after cancer breast surgery. Reg Anesth Pain Med 2001; 26: 223-8
158. Fassoulaki A, Triga A, Melemeni A, Sarantopoulos C: Multimodal analgesia with gabapentin and local anesthetics prevents acute and chronic pain after breast surgery for cancer. Anesth Analg 2005; 101: 1427-32
159. Moiniche S, Kehlet H, Dahl JB: A qualitative and quantitative systematic review of preemptive analgesia for postoperative pain relief: the role of timing of analgesia. Anesthesiology 2002; 96: 725-41
160. Ochroch EA, Gottschalk A, Augostides J, Carson KA, Kent L, Malayaman N, Kaiser LR, Aukburg SJ: Long-term pain and activity during recovery from major thoracotomy using thoracic epidural analgesia. Anesthesiology 2002; 97: 1234-44.
161. Reuben SS: Preventing the development of complex regional pain syndrome after surgery. Anesthesiology 2004; 101: 1215-24
162. Reuben SS, Makari-Judson G, Lurie SD: Evaluation of efficacy of the perioperative administration of venlafaxine XR in the prevention of postmastectomy pain syndrome. J Pain Symptom Manage 2004; 27: 133-9
163. Gianetta E, Cuneo S, Vitale B, Camerini G, Marini P, Stella M: Anterior tension-free repair of recurrent inguinal hernia under local anesthesia: a 7-year experience in a teaching hospital. Ann Surg 2000; 231: 132-6
164. Nikolajsen L, Ilkjaer S, Christensen JH, Kroner K, Jensen TS: Randomised trial of epidural bupivacaine and morphine in prevention of stump and phantom pain in lower-limb amputation. Lancet 1997; 350: 1353-7
165. Ong CK, Lirk P, Seymour RA, Jenkins BJ: The efficacy of preemptive analgesia for acute postoperative pain management: a meta-analysis. Anesth Analg 2005; 100: 757-73
166. Exadaktylos AK, Buggy DJ, Moriarty DC, Mascha E, Sessler DI: Can anesthetic technique for primary breast cancer surgery affect recurrence or metastasis? Anesthesiology 2006; 4: 660-4
167. Biki B, Mascha E, Moriarty DC, Fitzpatrick JM, Sessler DI, Buggy DJ: Anesthetic technique for radical prostatectomy surgery affects cancer recurrence: a retrospective analysis. Anesthesiology 2008; 109: 180-7
168. Sessler DI: Does regional analgesia reduce the risk of cancer recurrence? A hypothesis. Eur J Cancer Prev 2008; 17: 269-72
169. Sessler DI, Ben-Eliyahu S, Mascha EJ, Parat MO, Buggy DJ: Can regional analgesia reduce the risk of recurrence after breast cancer? Methodology of a multicenter randomized trial. Contemp Clin Trials 2008; 29: 517-26
170. Kurz A, Sessler DI, Lenhardt RA, Study of wound infections and temperature group: Perioperative normothermia to reduce the incidence of surgical-wound infection and shorten hospitalization. N Engl J Med 1996; 334: 1209-15
171. Sheffield CW, Sessler DI, Hunt TK: Mild hypothermia during isoflurane anesthesia decreases resistance to E. Coli dermal infection in guinea pigs. Acta Anaesthesiol Scand 1994; 38: 201-5
172. Sheffield CW, Sessler DI, Hunt TK, Scheuenstuhl H: Mild hypothermia during halothane anesthesia decreases resistance to S. Aureus dermal infection in guinea pigs. Wound Rep Reg 1994; 2: 48-56
173. Wenisch C, Narzt E, Sessler DI, Parschalk B, Lenhardt R, Kurz A, Graninger W: Mild intraoperative hypothermia reduces production of reactive oxygen intermediates by polymorphonuclear leukocytes. Anesth Analg 1996; 82: 810-6
174. Hynson J, Sessler DI: Intraoperative warming therapies: A comparison of three devices. J Clin Anesth 1992; 4: 194-9
175. Foley KM: The treatment of cancer pain. N Engl J Med 1985; 313: 84-95

176. Levy MH: Pharmacologic treatment of cancer pain. N Engl J Med 1996; 335: 1124-32
177. Cherny NI: Opioid analgesics: comparative features and prescribing guidelines. Drugs 1996; 51: 713-37
178. Anderson R, Saiers JH, Abram S, Schlicht C: Accuracy in equianalgesic dosing. Conversion dilemmas. J Pain Symptom Manage 2001; 21: 397-406
179. American Pain Society: Principles Of Analgesic Use In The Treatment Of Acute And Cancer Pain, Fifth Edition. Glenview, IL, American Pain Society, 2003
180. Moller JF, Nikolajsen L, Rodt SA, Ronning H, Carlsson PS: Thoracic paravertebral block for breast cancer surgery: a randomized double-blind study. Anesth Analg 2007; 105: 1848-51
181. Karmakar MK: Thoracic paravertebral block. Anesthesiology 2001; 95: 771-80
182. Ben-Eliyahu S, Page GG, Shakhar G, Taylor AN: Increased susceptibility to metastasis during pro-oestrus/oestrus in rats: possible role of oestradiol and natural killer cells. Br J Cancer 1996; 74: 1900-7
183. Page GG, Ben-Eliyahu S: Increased surgery-induced metastasis and suppressed natural killer cell activity during proestrus/estrus in rats. Breast Cancer Res Treat 1997; 45: 159-67
184. Rampil IJ: A primer for EEG signal processing in anesthesia. Anesthesiology 1998; 89: 980-1002.
185. Glass PSA, Bloom M, Kearse L, Rosow C, Sebel P, Manberg P: Bispectral analysis measures sedation and memory effects of propofol, midazolam, isoflurane and alfentanil in healthy volunteers. Anesthesiology 1997; 86: 836-47
186. Sebel PS, Lang E, Rampil IJ, White PF, Cork R, Jopling M, Smith NT, Glass PS, Manberg P: A multicenter study of bispectral electroencephalogram analysis for monitoring anesthetic effect. Anesth Analg 1997; 84: 891-9
187. Myles PS, Leslie K, McNeil J, Forbes A, Chan MT: Bispectral index monitoring to prevent awareness during anaesthesia: the B-Aware randomised controlled trial. Lancet 2004; 363: 1757-63
188. Gibb DB, Pikler N: Piritramide--a new long-acting analgesic. Anaesth Intensive Care 1973; 1: 308-14
189. Kay B: A clinical investigation of piritramide in the treatment of postoperative pain. Br J Anaesth 1971; 43: 1167-71
190. Paik S, Shak S, Tang G, Kim C, Baker J, Cronin M, Baehner FL, Walker MG, Watson D, Park T, Hiller W, Fisher ER, Wickerham DL, Bryant J, Wolmark N: A multigene assay to predict recurrence of tamoxifen-treated, node-negative breast cancer. N Engl J Med 2004; 351: 2817-26
191. Habel LA, Shak S, Jacobs MK, Capra A, Alexander C, Pho M, Baker J, Walker M, Watson D, Hackett J, Blick NT, Greenberg D, Fehrenbacher L, Langholz B, Quesenberry CP: A population-based study of tumor gene expression and risk of breast cancer death among lymph node-negative patients. Breast Cancer Res 2006; 8: R25
192. Kaklamani V: A genetic signature can predict prognosis and response to therapy in breast cancer: Oncotype DX. Expert Rev Mol Diagn 2006; 6: 803-9
193. Eden P, Ritz C, Rose C, Ferno M, Peterson C: "Good Old" clinical markers have similar power in breast cancer prognosis as microarray gene expression profilers. Eur J Cancer 2004; 40: 1837-41
194. Ware J, Jr., Kosinski M, Keller SD: A 12-Item Short-Form Health Survey: construction of scales and preliminary tests of reliability and validity. Med Care 1996; 34: 220-33
195. McDowell I: Measuring health : a guide to rating scales and questionnaires, 3rd Edition. Oxford ; New York, Oxford University Press, 2006
196. Cleeland CS, Ryan KM: Pain assessment: global use of the Brief Pain Inventory. Ann Acad Med Singapore 1994; 23: 129-38
197. Daut RL, Cleeland CS, Flanery RC: Development of the Wisconsin Brief Pain Questionnaire to assess pain in cancer and other diseases. Pain 1983; 17: 197-210
198. Backonja MM, Krause SJ: Neuropathic pain questionnaire--short form. Clin J Pain 2003; 19: 315-6
199. Krause SJ, Backonja MM: Development of a neuropathic pain questionnaire. Clin J Pain 2003; 19: 306-14
200. Nair VN: Confidence bands for survival functions with censored data: A comparitive study. Technometrics 1984; 14: 265-75
201. Hauck WW, Anderson S, Marcus SM: Should we adjust for covariates in nonlinear regression analyses of randomized trials? Control Clin Trials 1998; 19: 249-56
202. Ford I, Norrie J: The role of covariates in estimating treatment effects and risk in long-term clinical trials. Stat Med 2002; 21: 2899-908
203. Canner PL: Covariate adjustment of treatment effects in clinical trials. Control Clin Trials 1991; 12: 359-66

204. Pocock SJ, Assmann SE, Enos LE, Kasten LE: Subgroup analysis, covariate adjustment and baseline comparisons in clinical trial reporting: current practice and problems. Stat Med 2002; 21: 2917-30
205. Monk TG, Saini V, Weldon BC, Sigl JC: Anesthetic management and one-year mortality after noncardiac surgery. Anesth Analg 2005; 100: 4-10
206. Lennmarken C, Lindholm M, Greenwald S, Sandin R: Confirmation thatlow intraoperative BIS levels predict increased risk of post-operative mortality (abstract). Anesthesiology 2003; 99: A303
207. Rochon J: Accounting for Covariates Observed Post Randomization for Discrete and Continuous Repeated Measures Data. J R Statist Soc B 1996; 58: 205-19
208. Rochon J: Supplementing the Intent-to-Treat Analysis: Accounting for Covariates Observed Postrandomization in Clinical Trials to survival analysis. J Am Statist Assoc 1995; 90: 292-300
209. Saphner T, Tormey DC, Gray R: Annual hazard rates of recurrence for breast cancer after primary therapy. J Clin Oncol 1996; 14: 2738-46
210. Hwang IK, Shih WJ, De Cani JS: Group sequential designs using a family of type I error probability spending functions. Stat Med 1990; 9: 1439-45
211. Greif R, Akça O, Horn E-P, Kurz A, Sessler DI, Outcomes Research™ Group: Supplemental perioperative oxygen to reduce the incidence of surgical wound infection. N Engl J Med 2000; 342: 161-7
212. Fleischmann E, Lenhardt R, Kurz A, Herbst F, Fulesdi B, Greif R, Sessler DI, Akca O: Nitrous oxide and risk of surgical wound infection: a randomised trial. Lancet 2005; 366: 1101-7
213. Kabon B, Akca O, Taguchi A, Nagele A, Jebadurai R, Arkilic CF, Sharma N, Ahluwalia A, Galandiuk S, Fleshman J, Sessler DI, Kurz A: Supplemental intravenous crystalloid administration does not reduce the risk of surgical wound infection. Anesth Analg 2005; 101: 1546-53
214. Winkler M, Akça O, Birkenberg B, Hetz H, Scheck T, Arkilic CF, Kabon B, Marker E, Grubl A, Czepan R, Greher M, Goll V, Gottsauner-Wolf F, Kurz A, Sessler DI: Aggressive warming reduces blood loss during hip arthroplasty. Anesth Analg 2000; 91: 978-84
215. Schmied H, Kurz A, Sessler DI, Kozek S, Reiter A: Mild intraoperative hypothermia increases blood loss and allogeneic transfusion requirements during total hip arthroplasty. Lancet 1996; 347: 289-92
216. Lenhardt R, Marker E, Goll V, Tschernich H, Kurz A, Sessler DI, Narzt E, Lackner F: Mild intraoperative hypothermia prolongs postanesthetic recovery. Anesthesiology 1997; 87: 1318-23
217. Sessler DI, Rubinstein EH, Moayeri A: Physiological responses to mild perianesthetic hypothermia in humans. Anesthesiology 1991; 75: 594-610
218. Kurz A, Sessler DI, Narzt E, Bakar A, Lenhardt R, Huemer G: Postoperative hemodynamic and thermoregulatory consequences of intraoperative core hypothermia. J Clin Anesth 1995; 7: 359-66
219. Belda FJ, Aguilera L, Garcia de la Asuncion J, Alberti J, Vicente R, Ferrandiz L, Rodriguez R, Company R, Sessler DI, Aguilar G, Botello SG, Orti R: Supplemental perioperative oxygen and the risk of surgical wound infection: a randomized controlled trial. JAMA 2005; 294: 2035-42
220. Koch CG, Li L, Sessler DI, Figueroa P, Hoeltge GA, Mihaljevic T, Blackstone EH: Duration of red-cell storage and complications after cardiac surgery. N Engl J Med 2008; 358: 1229-39
221. Shafer SL, Gregg KM: Algorithms to rapidly achieve and maintain stable drug concentrations at the site of drug effect with a computer-controlled infusion pump. J Pharmacokinet Biopharm 1992; 20: 147-69
222. Meinert CL: Clinical Trials: Design, Conduct, and Analysis. New York, Oxford University Press, 1986
223. Fisher B, Anderson S, Bryant J, Margolese RG, Deutsch M, Fisher ER, Jeong JH, Wolmark N: Twenty-year follow-up of a randomized trial comparing total mastectomy, lumpectomy, and lumpectomy plus irradiation for the treatment of invasive breast cancer. N Engl J Med 2002; 347: 1233-41
224. Rahim RT, Meissler JJ, Zhang L, Adler MW, Rogers TJ, Eisenstein TK: Withdrawal from morphine in mice suppresses splenic macrophage function, cytokine production, and costimulatory molecules. J Neuroimmunol 2003; 144: 16-27
225. Page GG, McDonald JS, Ben-Eliyahu S: Pre-operative versus postoperative administration of morphine: impact on the neuroendocrine, behavioural, and metastatic-enhancing effects of surgery. Br J Anaesth 1998; 81: 216-23
226. Page GG, Ben-Eliyahu S, Liebeskind JC: The role of LGL/NK cells in surgery-induced promotion of metastasis and its attenuation by morphine. Brain Behav Immun 1994; 8: 241-50
227. Page GG, Ben-Eliyahu S, Yirmiya R, Liebeskind JC: Morphine attenuates surgery-induced enhancement of metastatic colonization in rats. Pain 1993; 54: 21-8

228. D'Ambrosio A, Borghi B, Damato A, D'Amato G, Antonacci D, Valeri F: Reducing perioperative blood loss in patients undergoing total hip arthroplasty. Int J Artif Organs 1999; 22: 47-51
229. O'Hara DA, Duff A, Berlin JA, Poses RM, Lawrence VA, Huber EC, Noveck H, Strom BL, Carson JL: The effect of anesthetic technique on postoperative outcomes in hip fracture repair. Anesthesiology 2000; 92: 947-57.

I. Consortium/Contractual Arrangements

Our study will require enrolling a total of 1,100 patients undergoing mastectomy over a five-year period (≈250/year). Even at the Cleveland Clinic, we would not be able to recruit so many patients. We thus propose a multi-center trial with six universities participating in addition to the main Cleveland Clinic site. There is also one investigator at the University of Tel Aviv, although Tel Aviv will not be a clinical site for the trial. As might thus be expected, we have consortium agreements with the University of Tel Aviv and the six other universities, each of which has a designated Site Director who will take responsibility for patient enrollment and data acquisition. Trial management will nonetheless be centralized at the Cleveland Clinic and the Study Chair (PI) will take overall responsibility for the study, including monitoring and auditing.

J. Resource Sharing

Upon completion of the trial and publication of the main results that will be provided to PubMed Central, the master dataset will be de-identified to meet HIPAA requirements. The resulting limited dataset will be published on the **O**utcomes **R**esearch website (www.or.org). De-identified versions of the study database will also be provided to interested investigators to encourage secondary analyses.
